# Gut microbiota-derived extracellular vesicles in Parkinson’s disease

**DOI:** 10.1016/j.isci.2026.117057

**Published:** 2026-08-05

**Authors:** Sonja Karikka, Mysore V. Tejesvi, Jenni Hekkala, Jenni Turunen, Anna Kaisanlahti, Nikke Virtanen, Surbhi Mishra, Artem Zhyvolozhnyi, Nelli Sinkkonen, Marko Suokas, Sanna Meriläinen, Pande Putu Erawijantari, Leo Lahti, Juha Saarnio, Johanna Krüger, Anne M. Portaankorva, Seppo J. Vainio, Terhi Ruuska-Loewald, Justus Reunanen, Anatoliy Samoylenko

**Affiliations:** 1 Research Unit of Translational Medicine, University of Oulu, Oulu, Finland; 2 Biocenter Oulu, University of Oulu, Oulu, Finland; 3 Laboratory of Developmental Biology, Disease Networks Research Unit, Faculty of Biochemistry and Molecular Medicine, University of Oulu, Oulu, Finland; 4 Kvantum Institute, University of Oulu, Oulu, Finland; 5 Research Unit of Clinical Medicine, University of Oulu, Oulu, Finland; 6 Department of Pediatrics and Adolescent Medicine, Oulu University Hospital, Oulu, Finland; 7 Department of Computing, University of Turku, Turku, Finland; 8 Research Unit of Clinical Medicine, Neurology, University of Oulu, Oulu, Finland; 9 Medical Research Center, Oulu University Hospital, Oulu, Finland; 10 Neurocenter, Neurology, Oulu University Hospital, Oulu, Finland; 11 Clinical Neurosciences, University of Helsinki, Helsinki, Finland

**Keywords:** Parkinson’s disease, gut microbiota, microbiota-gut-brain axis, bacterial extracellular vesicles, 16S rRNA gene sequencing, proteome

## Abstract

The gut microbiota contributes to the pathophysiology of Parkinson’s disease (PD), but the mechanism is not known. Extracellular vesicles (EVs), membrane-enclosed nanosized particles, contain various biomolecules, cross biological barriers, and reach sterile body compartments. We hypothesized that EVs released by the gut microbiota might influence PD pathophysiology. We isolated gut microbiota-derived EVs from the fecal samples of 20 patients with PD and 19 healthy controls. Patients with PD had higher concentrations of fecal EVs, and the mean particle size of EVs was larger. The 16S ribosomal RNA analysis of gut microbiota and microbiota-derived EVs showed taxonomic differences at both the phylum and genus levels between patients with PD and healthy controls. Proteomic analysis of EVs identified unique protein signatures in patients with PD. These findings suggest that gut microbiota-derived EVs constitute a disease-relevant compartment in PD, with their molecular cargo representing a potential mechanistic link between gut microbiota and PD.

## Highlights

• Gut microbiota-derived extracellular vesicles may contribute to Parkinson’s disease
• Gut microbiota and vesicle origin were different in the Parkinson’s disease group
• Loss of the predicted protective potential of vesicles in the Parkinson’s disease group
• Vesicle proteome was different in the Parkinson’s disease group

## Graphical abstract

**Figure:**
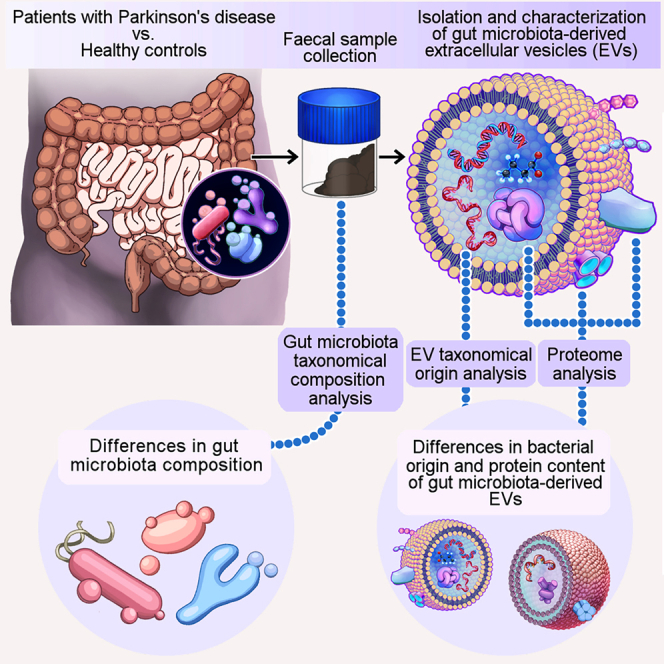

## Teaser

Health sciences; Medicine; Medical specialty; Psychiatry; Internal medicine; Endocrinology; Gastroenterology; Neurology

## Introduction

Parkinson’s disease (PD) is the second most common neurodegenerative disease, characterized by loss of dopaminergic neurons in the substantia nigra pars compacta area of the brain and accumulation of misfolded α-synuclein.^1^ Prodromal gastrointestinal symptoms can predate the diagnosis of PD by as much as a decade or more, which suggests that PD might originate from the gut.^2^^,^^3^^,^^4^^,^^5^ Furthermore, the gut microbiota has been associated with PD pathophysiology.^6^^,^^7^^,^^8^^,^^9^ Patients with prodromal PD symptoms and patients with PD have shown similar changes in gut microbiota and metagenomic pathways compared to healthy controls.^10^ Altered functionality and balance of gut microbial community have been reported in patients with PD,^11^ accompanied by a shift toward a pro-inflammatory state and reduced neuroprotective capacity.^12^^,^^13^ Moreover, changes in circulating metabolites have been linked to changes in gut microbiota composition and disease severity in patients with PD.^14^^,^^15^^,^^16^^,^^17^

It has been proposed that the gut microbiota influences PD development by transporting pathogenic inducers via a neuronal route^18^ or blood circulation.^18^^,^^19^ However, the exact mechanism is not known. One potential factor is extracellular vesicles (EVs) secreted by the gut microbiota. EVs are membrane-enclosed nanosized particles, secreted by both gram-positive and gram-negative bacteria.^20^^,^^21^ Bacterial EVs, containing different biomolecules including proteins, lipids, metabolites, and nucleic acids, have multiple functions related to bacterial survival and bacteria-host interactions.^22^ Interestingly, gut microbiota-derived EVs have been found in usually sterile biofluids such as blood^23^ and amniotic fluid.^24^ Furthermore, bacterial EVs can cross physiological barriers including the intestinal epithelium and the blood-brain barrier, which might enable bacterial influence on the host without actual cell-to-cell contact.^25^^,^^26^^,^^27^^,^^28^ It has been suggested that EVs secreted by gut bacteria may carry toxic or inflammatory components into the brain,^29^ but the role of gut microbiota-derived EVs in the development and progression of PD has not been studied. We hypothesized that gut microbiota-derived EVs and their cargo are altered in patients with PD and may have a role in PD pathophysiology.

In this controlled clinical study, we set out to investigate gut microbiota-derived EVs from fecal samples of healthy controls (*n* = 19) and patients with PD (*n* = 20). Our aim was to compare whether gut microbiota-derived EVs in patients with PD have a distinct taxonomic origin and protein cargo compared to healthy controls.

## Results

### Size and concentration of gut microbiota-derived extracellular vesicles differs between healthy controls and patients with Parkinson’s disease

**Figure 1:**
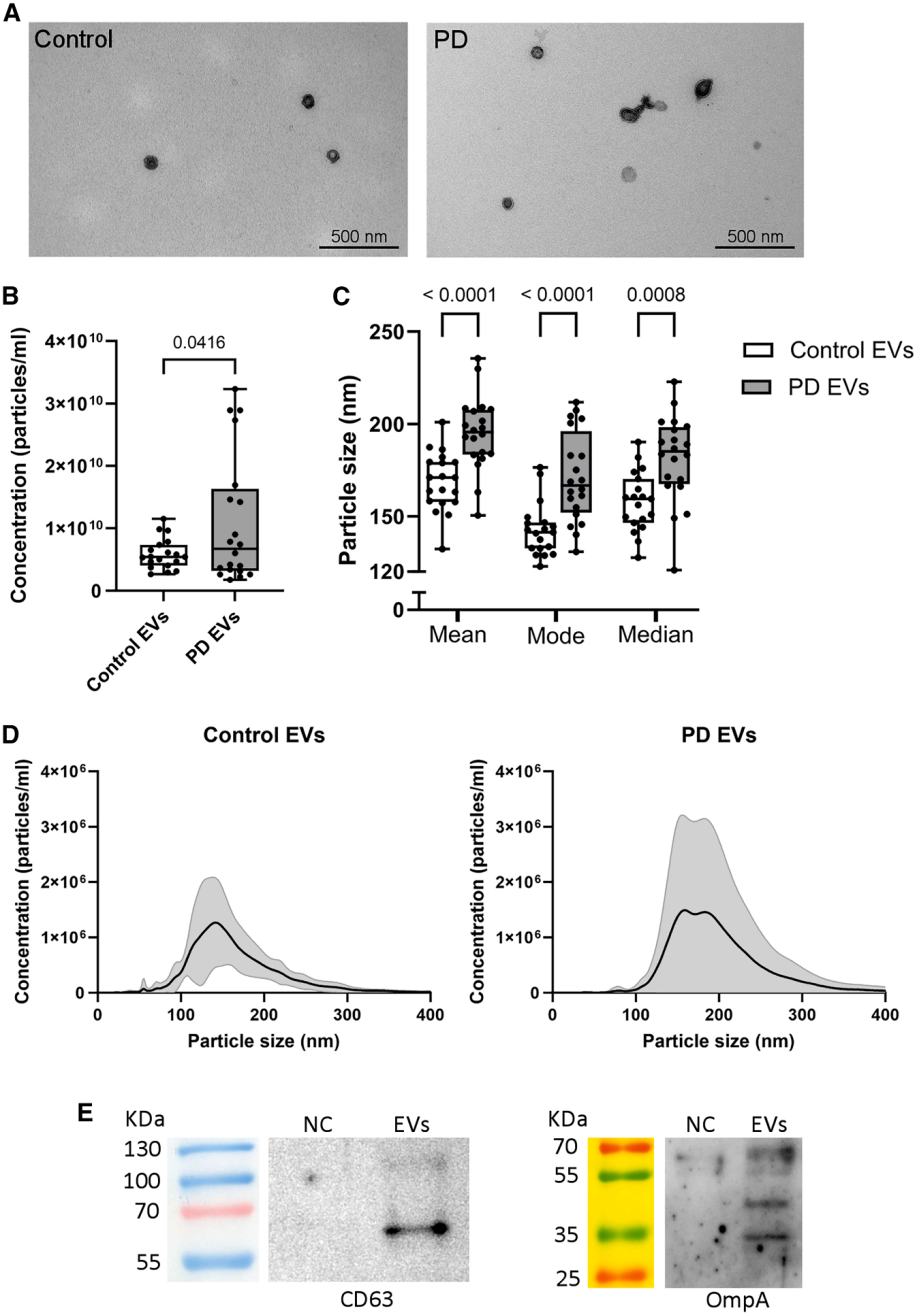
Characterization of gut microbiota-derived EVs using TEM, NTA, and western blot (A) Representative TEM images (23,000 × magnification, 500 nm scale bars) of EVs from healthy controls and patients with PD (uncropped images in Figures S1A and S1B). (B) Mean concentration (particles/mL, *p* = 0.0416, unpaired *t* test with Welch’s correction) of individual gut microbiota-derived EV samples (healthy controls = 19, PD = 20). (C) Mean (194 ± 19 vs. 168 ± 15 nm, *p* < 0.0001), mode (169 ± 24 vs. 142 ± 14 nm, *p* < 0.0001), and median (180 ± 23 vs. 157 ± 16 nm, *p* = 0.0008) size of EVs was higher in patients with PD compared to healthy controls. Unpaired *t* test *p* values are shown. (D) Size distribution of gut microbiota-derived EVs. The black line corresponds to mean size and the gray area represents standard deviation. (E) Western blot images of EV samples probed with antibodies against human exosome marker CD63 and bacterial outer membrane vesicle marker OmpA (uncropped images in Figures S2A and S2B). NC: negative control that was carried through EV isolation process.

Gut microbiota-derived EVs were isolated from 19 healthy controls and 20 patients with PD. The presence of EVs was confirmed with transmission electron microscopy (TEM) (Figure 1A). Nanoparticle tracking analysis (NTA) showed that concentration of EVs varied from 10^9^ to 10^10^ particles/mL per 1 g of fecal material in both groups (Figure 1B). Higher concentrations of EVs (particles/mL ± SD) were observed in patients with PD compared to healthy controls (7.19 × 10^9^ ± 1.01 × 10^10^ vs. 5.42 × 10^9^ ± 2.38 × 10^9^ particles/mL, *p* = 0.042). Mean, mode, and median particle size (nm ± SD) were significantly larger in patients with PD compared to those of healthy controls (Figure 1C). The PD group showed greater variation in EV size than the control group (Figure 1D). The presence of markers typical for human exosomes (CD63) and bacterial outer membrane vesicles (OmpA) was confirmed by western blot (Figure 1E; Figures S2A and S2B).

### 16S rRNA analysis of fecal samples and gut microbiota-derived extracellular vesicles from healthy controls and patients with Parkinson’s disease shows distinctive features of bacterial composition

**Figure 2:**
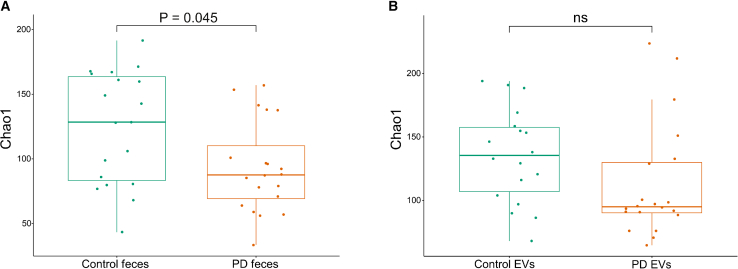
Alpha diversity of gut microbiota and gut microbiota-derived EVs The alpha diversity of fecal and EV samples was determined using the Chao1 index. Significance was tested using Kruskal-Wallis H test with Benjamini-Hochberg correction. (A) Alpha diversity of gut microbiota in healthy controls (*n* = 19) and patients with PD (*n* = 20). (B) Alpha diversity of gut microbiota-derived EVs in healthy controls (*n* = 18) and patients with PD (*n* = 20). ns: not significant.

Within-sample diversity i.e., alpha diversity of bacteria taxa present in the gut microbiota and gut microbiota-derived EVs was assessed using the Chao1 index, number of observed features and Shannon index. Significance was determined using the Kruskal-Wallis H test and *p* values were adjusted with Benjamini-Hochberg correction. Alpha diversity was lower (*p* = 0.045) in the fecal samples of patients with PD (Figure 2A; Figures S3A and S3C) but the difference in taxonomic composition of EV origin was not statistically significant (Figure 2B; Figures S3B and S3D).

**Figure 3:**
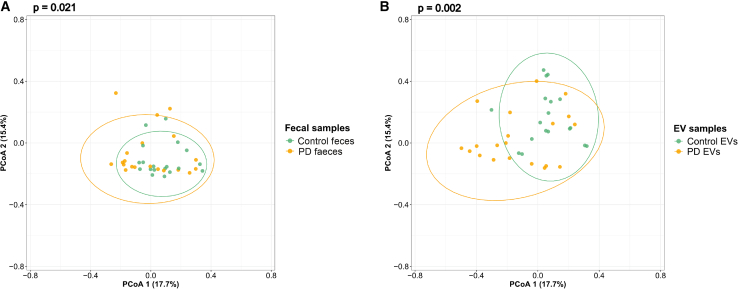
PCoA of gut microbiota and taxonomic composition of EV origin The Bray-Curtis dissimilarity index was used in comparisons. Significance was tested using PERMANOVA. (A) Beta-diversity of gut microbiota differed significantly (*p* = 0.021) between healthy controls (*n* = 19) and patients with PD (*n* = 20). (B) Beta-diversity of taxonomic composition of EV origin differed significantly (*p* = 0.002) between healthy controls (*n* = 18) and patients with PD (*n* = 20).

Then, between-sample diversity i.e., the beta diversity of bacterial populations from healthy controls and patients with PD was assessed using principal coordinate analysis (PCoA) with the Bray-Curtis dissimilarity index and tested for significance using PERMANOVA. The analysis was performed at the genus level. Both gut microbiota (Figure 3A) and taxonomic composition of EV origin (Figure 3B) formed distinct clusters in the control and PD groups (*p* = 0.021 for gut microbiota, *p* = 0.002 for EVs).

**Figure 4:**
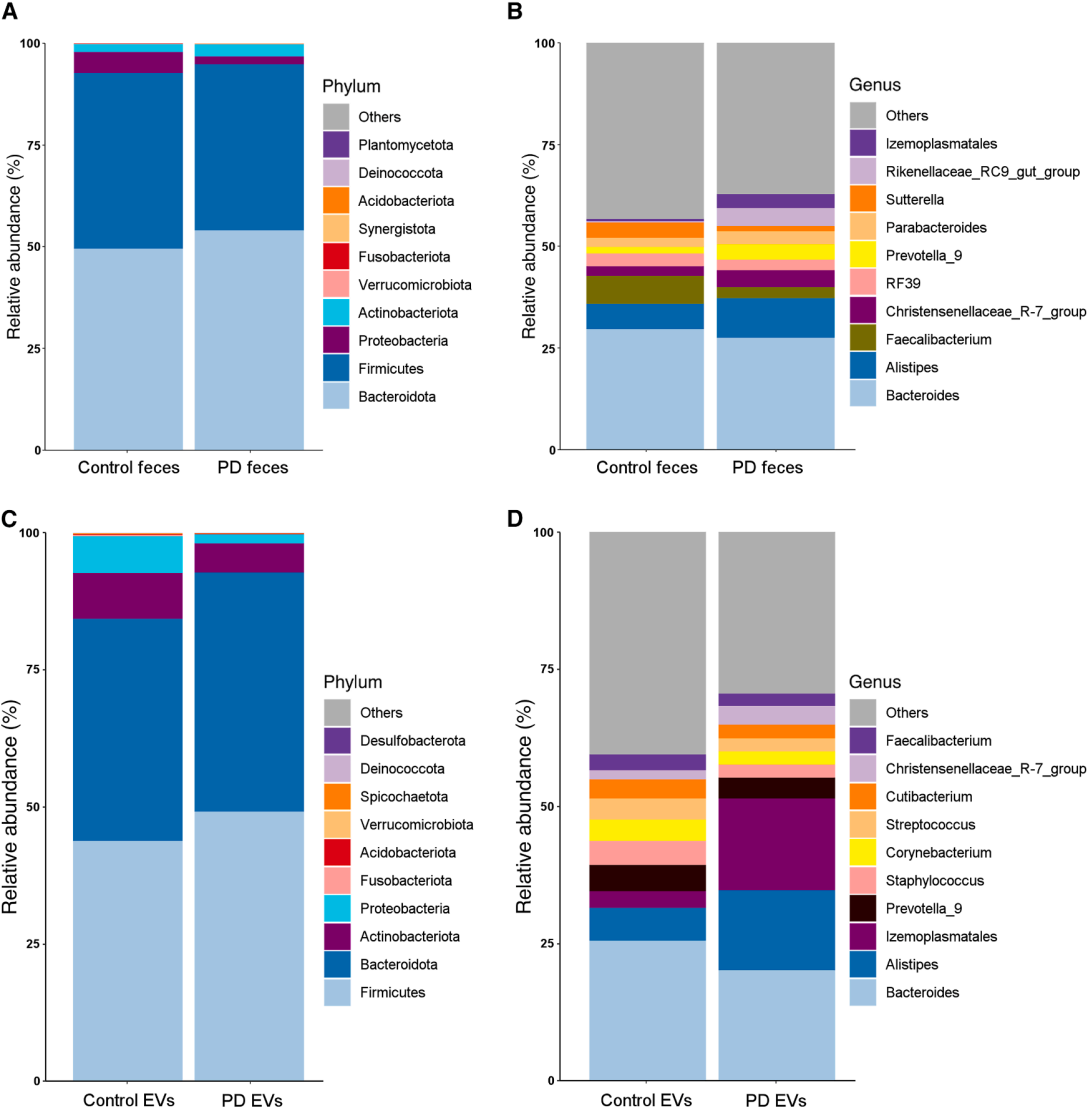
Relative abundance of phyla and genera found in feces and gut microbiota-derived EVs (A) Phylum-level gut microbiota composition in healthy controls (*n* = 19) and patients with PD (*n* = 20). (B) Genus-level gut microbiota composition in healthy controls and patients with PD. (C) Phylum-level taxonomic composition of EV origin in feces of healthy controls (*n* = 18) and patients with PD (*n* = 20). (D) Genus-level taxonomic composition of EV origin in feces of healthy controls and patients with PD.

Next, the taxonomic composition of gut microbiota in fecal samples at the phylum and genus levels (Figures 4A and 4B; Figures S4A and S5A; Tables S2 and S3) and taxonomic composition of EV origin was compared to receive an overall view of the taxa present in the study groups (Figures 4C and 4D; Figures S4B and S5B; Tables S2 and S3).

**Figure 5:**
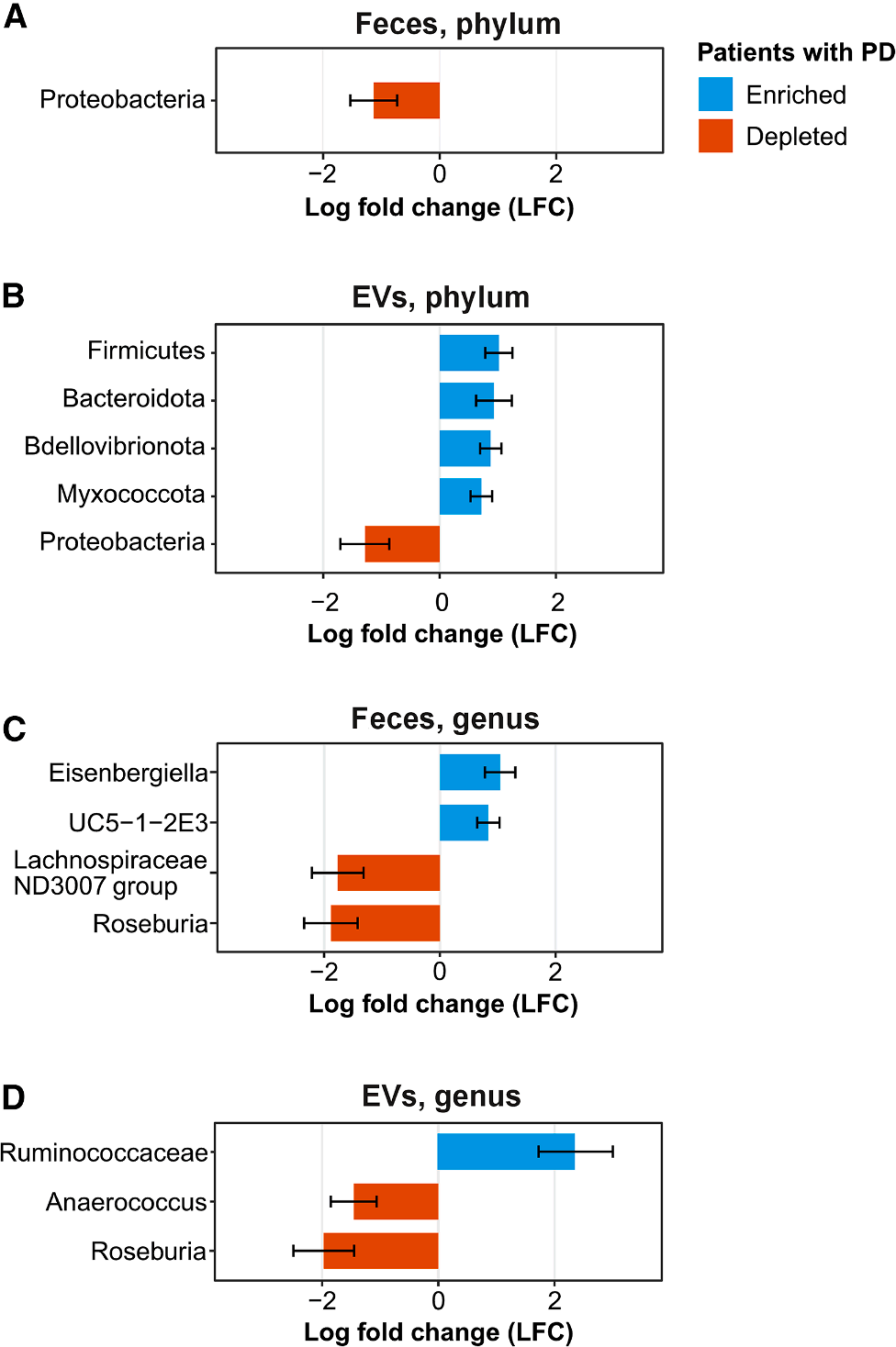
Differential abundance analysis using ANCOM-BC The analysis was performed for the gut microbiota of fecal samples and taxonomic composition of EV origin in feces of healthy controls (19 fecal samples and 18 EV samples) and patients with PD (20 fecal and EV samples). The figures show statistically significant (*p* < 0.05 in ANCOM-BC analysis with Bonferroni correction) enrichment and depletion of bacterial taxa relative to patients with PD. The *x* axis shows the log fold change. The *y* axis shows the feature IDs at phylum (A and B) and genus level (C and D). Error bars indicate standard error.

Then, differentially abundant bacteria taxa present in the fecal samples and EV samples were analyzed using ANCOM-BC. In the fecal samples, depletion of Proteobacteria was found in the gut microbiota of patients with PD as compared to the control group (Figure 5A). In the gut-microbiota-derived EVs of patients with PD, taxonomic composition of EV origin showed enrichment of phyla Firmicutes, Bacteroidota, Bdellovibrionota, and Myxococcota and depletion of Proteobacteria as compared to control samples (Figure 5B). At the family and genus levels, the genera *Eisenbergiella* and UC5-1-2E3 were enriched and the Lachnospiraceae_ND3007 group and *Roseburia* were depleted in the gut microbiota of patients with PD (Figure 5C). Taxonomic composition of EV origin in feces showed that an unidentified genus belonging to the family Ruminococcaceae was enriched and the genera *Anaerococcus* and *Roseburia* were depleted in patients with PD (Figure 5D).

**Figure 6:**
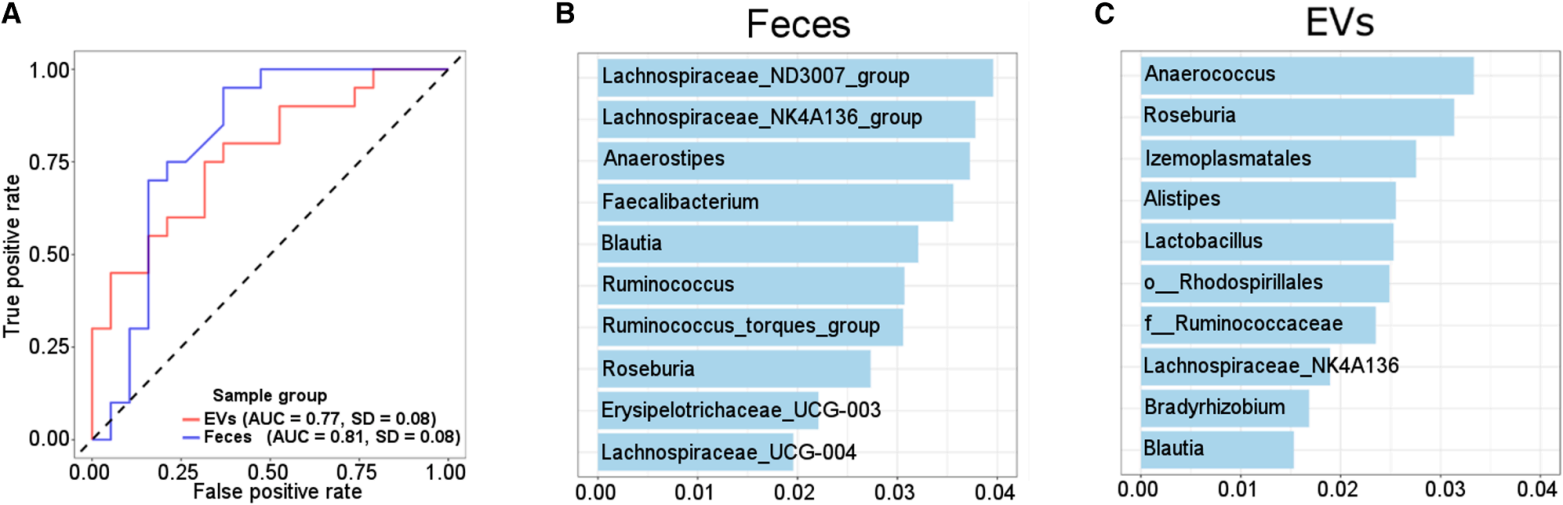
Machine learning classification using gut microbiota composition and taxonomic composition of EV origin (A) Receiver operating characteristics of the EV (red) and fecal (blue) sample groups (healthy controls and patients with PD) with false positive rate on the *x* axis and true positive rate on the *y* axis. The dotted line represents random chance. The classifier was trained using a Random Forest (RF) nested cross-validation algorithm. (B) Ten most important taxa for classifying fecal samples of healthy controls (*n* = 19) and patients with PD (*n* = 20). Importance value indicated on *x* axis. (C) Ten most important taxa for classifying gut microbiota-derived EV samples isolated from fecal samples from healthy controls (*n* = 18) and patients with PD (*n* = 20). AUC: area under curve, SD: standard deviation.

Machine learning approach was then used to further investigate compositional signatures present in the gut microbiota and gut microbiota-derived EVs. Machine learning classifiers, built using a random forest algorithm based on taxonomic profiling data from 16S rRNA sequencing, were able to distinguish patients with PD from healthy controls based on both the gut microbiota of fecal samples (Figures 6A and 6B) and taxonomic composition of EV origin in feces (Figure 6C), with an area under the curve (AUC) of 0.81 ± 0.08 for gut microbiota samples and 0.77 ± 0.08 for gut microbiota-derived EVs (Figure 6A). The most important features of the machine learning model were different in the gut microbiota and EV datasets (Figures 6B and 6C).

**Figure 7:**
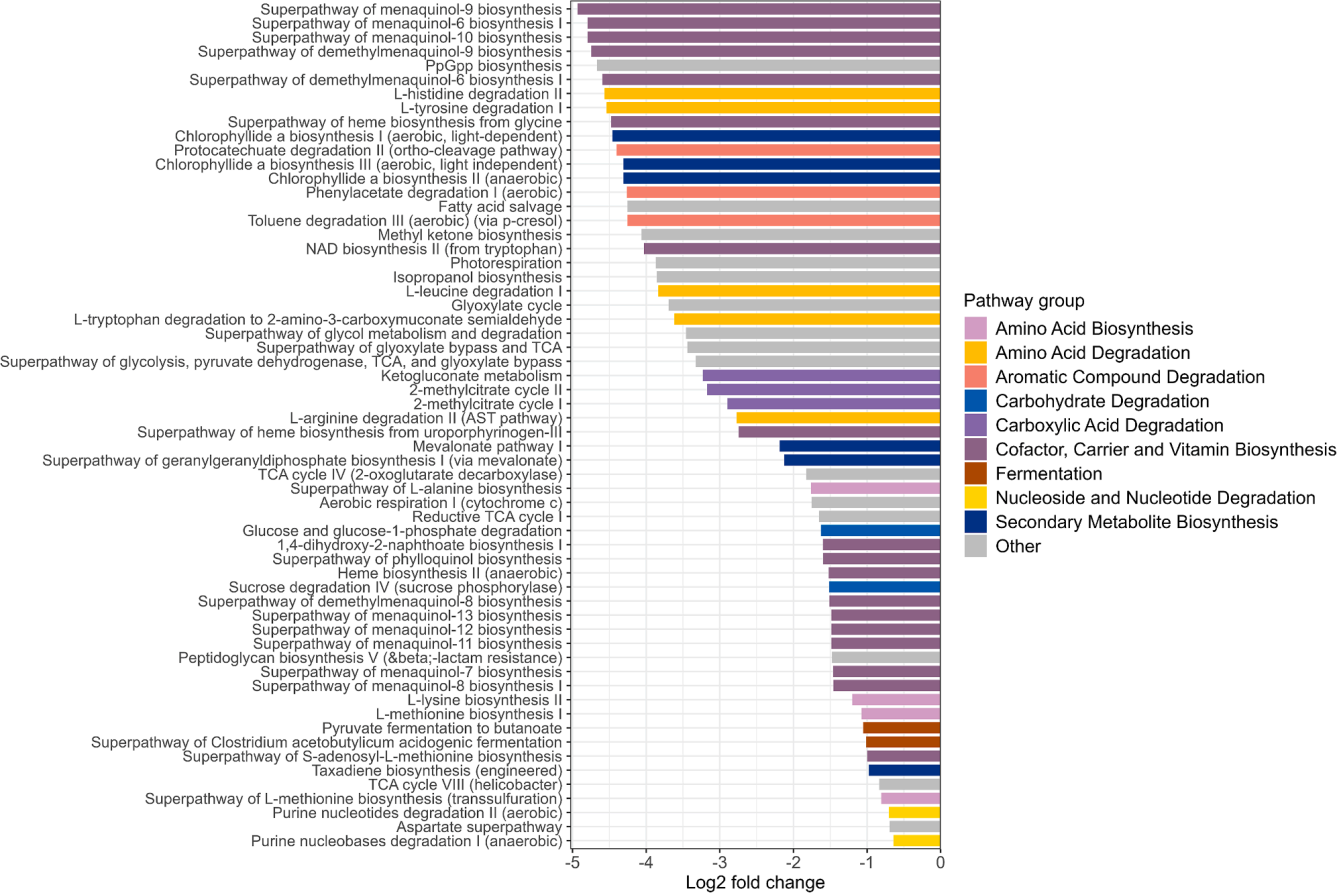
The differentially abundant predicted metabolic pathways in gut microbiota-derived EV samples between healthy controls and patients with PD MetaCyc abundances were compared between patients with PD (*n* = 20) and control samples (*n* = 18). Differential abundance analysis was performed using LinDA with FDR-adjustment for *p* values. Pathways with *p* < 0.001 are shown in the figure. Color indicates a group to which pathway belongs. Full list of differentially abundant pathways (*p* < 0.05) and mean relative abundances is provided in Data S1.

In order to have functional insight into gut microbiota and gut microbiota-derived EVs in PD, PICRUSt2 was used to predict metabolic pathways of both gut microbiota and gut microbiota-derived EVs using 16S rRNA sequencing data. In fecal samples, none of the pathways differed significantly. In EV samples, 130 differentially abundant (*p* < 0.05) pathways were found (Data S1) and 60 of them were highly significant (*p* < 0.001) (Figure 7). All differentially abundant pathways were depleted in patients with PD. The largest depleted pathway groups were related to cofactor, carrier and vitamin biosynthesis (20%, fold change range 1.9–30.5), aromatic compound degradation (13.8%, fold change range 4.3–21.1), amino acid degradation (7.7%, fold change range 2.3–23.4), secondary metabolite biosynthesis (5.4%, fold change range 2.0–22), amino acid biosynthesis (4.6%, fold change range 1.5–15.6), carbohydrate degradation (4.6%, fold change range 2.9–13.7), and carboxylic acid degradation (4.6%, fold change range 5.4–9.4) (Data S1). The highest fold changes (>20) for individual pathways were observed in biosynthesis of menaquinones, guanosine tetraphosphate (ppGpp) biosynthesis, histidine and tyrosine degradation, heme biosynthesis from glycine, chlorophyllide a biosynthesis, protocatechuate degradation, and cob(II)yrinate a,c-diamide biosynthesis (Data S1, Figure 7).

### Patients with Parkinson’s disease showed differences in the protein cargo of gut microbiota-derived EVs

**Figure 8:**
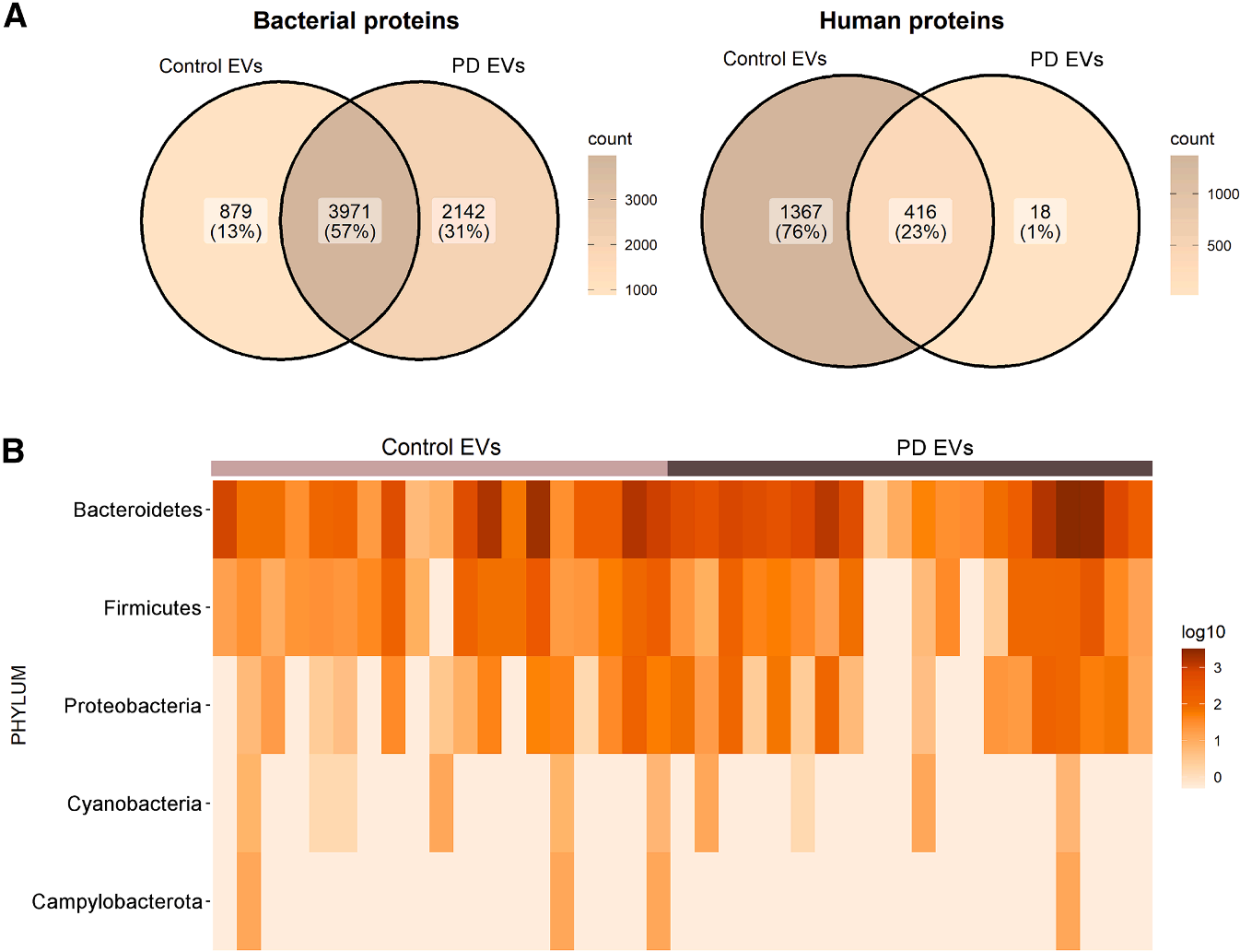
Protein distribution in gut microbiota-derived EVs between sample groups (A) Venn diagram comparing the numbers of unique bacteria and human proteins found in gut microbiota-derived EVs from healthy controls (*n* = 19) and patients with PD (*n* = 20). (B) Heatmap with base-10 logarithmic scaling of the most prominent bacterial phyla associated with observed proteins hits.

Next, the protein cargo of gut microbiota-derived EVs enriched for bacterial EVs during isolation was analyzed. A total of 4,850 unique bacterial proteins and 1,783 unique human proteins were identified in the EVs of healthy controls. In the EVs of patients with PD, 6,113 unique bacterial proteins and 434 unique human proteins were found. The mean number of bacterial proteins found in the EV samples of individual healthy controls was 569 (range 14–2,403) and 793 (range 2–3,478) in patients with PD. The mean number of human proteins was 147 (range 9–1,569) in healthy controls and 79 (range 30–148) in patients with PD. The bacteria-to-human protein ratio of protein cargo in EVs was 3:1 in healthy controls and 14:1 in patients with PD. In total, 13% (879) of the unique bacterial proteins found were present only in healthy controls and 31% (2,142) in patients with PD (Figure 8A). Relatively, 76% of the identified human proteins (1,367) were present in healthy controls, and 1% (18) were present in patients with PD (Figure 8A). The potential taxonomic origin of bacterial proteins was analyzed. Three phyla, Bacteroidetes (Bacteroidota), Firmicutes (Bacillota), and Proteobacteria (Pseudomonadota), had the highest number of associated protein hits (Figures 8B and S6).

**Figure 9:**
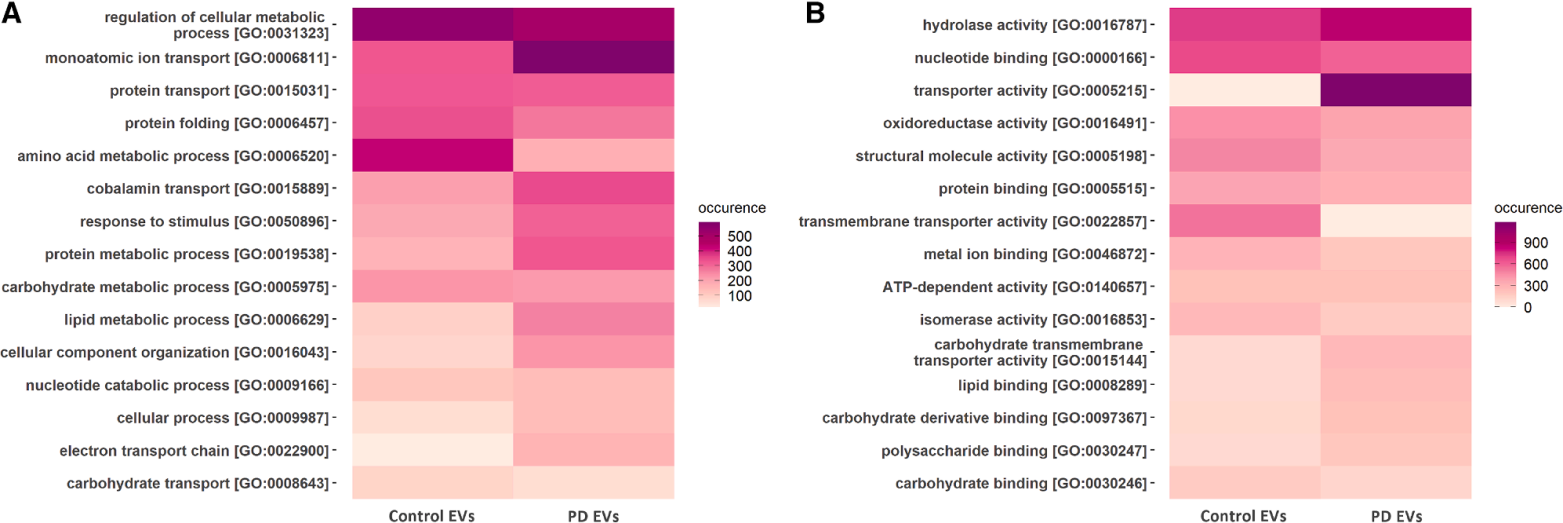
Top 15 most prominent bacterial protein GO annotations found in gut microbiota-derived EVs (A) Biological processes. (B) Molecular function. PD: Parkinson’s disease. EV samples included 19 samples from healthy controls and 20 samples from patients with PD.

To compare the functions of the gut microbiota-derived EV proteins of healthy controls and patients with PD, proteins were categorized by their gene ontology (GO) annotations according to their roles in biological processes and molecular function (Tables S4 and S5). The most common GO classes for biological processes in healthy controls included regulation of cellular metabolic process (10%), amino acid metabolic process (8%), and protein folding (6%), while in patients with PD monoatomic ion transport (13%), regulation of cellular metabolic process (11%), and cobalamin transport (8%) were the most prominent (Figures 9A and 9B). The most abundant GO classes for molecular function in healthy controls and patients with PD were similar: hydrolase activity (15% and 16%), nucleotide binding (15% and 11%) and transporter or transmembrane transporter activity (11% and 21%).

## Discussion

This controlled clinical study shows that the physical properties, taxonomical composition, and protein cargo of EVs secreted by gut microbiota differ between patients with PD and healthy controls. While our results are in line with previous research on the gut microbiota and gut-brain axis in PD pathogenesis,^30^ we extend the research on gut microbiota-host interaction through EVs. Our data suggest that interkingdom signaling between gut microbiota and host through EVs is different in healthy controls and patients with PD.

How the gut microbiota contributes to PD pathogenesis remains an active area of investigation.^31^ This study examines EVs secreted by the gut microbiota as a potential mediator of gut-brain signaling in PD. Most previous studies investigating bacterial EVs in chronic neurological diseases have used single strain bacterial cultures or animal models.^29^^,^^32^^,^^33^^,^^34^^,^^35^^,^^36^^,^^37^^,^^38^^,^^39^^,^^40^^,^^41^^,^^42^^,^^43^^,^^44^^,^^45^^,^^46^ In murine PD models, administration of EVs from *E*. *coli* using colonic injection have been shown to induce alpha-synuclein accumulation,^45^ whereas oral administration of *L*. *acidophilus* EVs exhibit neuroprotective effects.^46^ Studies focusing on Alzheimer’s disease have shown that the blood carries bacteria-derived EVs in a mouse model of Alzheimer’s disease^33^ and the gut microbiota-derived EVs of patients with Alzheimer’s disease promote Alzheimer’s disease-like features such as neuroinflammation, cognitive impairment, and Tau protein phosphorylation in mice.^29^^,^^42^^,^^43^ We recently showed that bacterial EVs reach the fetal environment, traditionally considered a sterile body compartment, in healthy pregnancy.^24^ In the present study, we did not investigate whether bacterial EVs reach the brain. Our results suggest, however, that the gut microbiota may communicate with the host through the different microbiota-derived EVs in patients with PD as compared to healthy controls. This finding is potentially important because previous studies have shown that bacterial EVs reach the brain through blood circulation and the vagus nerve, and induce neuronal pathologies such as neuroinflammation, neurotoxicity, cognitive impairment, depressive behavior, and amyloid β accumulation in mice^34^^,^^35^^,^^36^^,^^37^^,^^38^^,^^39^^,^^40^^,^^41^ and zebrafish.^44^ Bacterial EVs have been reported to cross the blood-brain barrier using both paracellular pathways *in vitro*^47^ and transcellular pathways *in vivo*.^48^ Furthermore, bacterial EVs have been shown to increase the permeability of the blood-brain barrier by down-regulating the expression of tight junction proteins *in vivo*^29^^,^^40^^,^^42^ and *in vitro*.^49^^,^^50^ Gut microbiota-derived EVs of patients with Alzheimer’s disease decreased the expression of tight junction proteins in mice more profoundly compared to those from healthy controls highlighting the importance of gut microbiota-derived EVs in brain-related pathologies.^29^^,^^42^

Our findings show significant differences in beta diversity in the gut microbiota of patients with PD in line with previous studies.^10^^,^^13^^,^^51^^,^^52^ Furthermore, here we expand on previous findings as we show significant differences in the taxonomic composition of EV origin in feces between healthy controls and patients with PD. Specifically, we found enrichment of *Ruminococcaceae* and depletion of *Roseburia* as the bacterial source of fecal EVs of patients with PD, which is in line with previous studies.^10^^,^^13^^,^^51^^,^^52^^,^^53^^,^^54^ The differences found in abundances between gut microbiota and taxonomic origin of EVs were not the same. It is likely that the bacterial origin of EVs mirrors the taxonomy of gut microbiota only partially. Differences in pathways observed in EVs, but not in the gut microbiota of patients with PD, further supports this idea. Thus, research focusing on gut microbiota-secreted EVs, including their cargo, is needed to investigate the pathogenesis of PD.

*Roseburia* was depleted both in the gut microbiota and as a bacterial source of gut microbiota-derived EVs in patients with PD in the present study. Depletion of *Roseburia* has been shown to be a consistent feature in the gut microbiota of patients with PD.^10^^,^^13^^,^^51^^,^^52^^,^^53^^,^^54^ In a longitudinal study conducted over three years, it was shown that a lower abundance of *Roseburia* was associated with a faster decline in motor, non-motor and cognitive functions in patients with PD.^55^ Members of the genus *Roseburia* are well known producers of short-chained fatty acids (SCFAs).^56^ Reduction in SCFA levels has previously been observed in patients with PD.^57^^,^^58^ Based on our results, depletion of EVs secreted by *Roseburia* might be associated with PD pathogenesis as well.

PD has been shown to be associated with alterations in the blood-brain barrier^59^ and intestinal permeability.^60^ Compromised barrier function together with depletion of beneficial bacteria and their EVs could promote the transfer of potentially detrimental gut microbiota EVs to the enteric nervous system or to the brain. Multiomic analysis of PD gut microbiota together with serum metabolites showed an association between *Eisenbergiella* and serum lipid metabolism related to sphingolipids.^14^ Sphingolipids are associated with α-synuclein aggregation and mitochondrial dysfunction related to PD.^61^^,^^62^ Our results show that the number of gut microbiota-derived EV proteins related to lipid metabolism was higher in patients with PD along with enrichment of *Eisenbergiella*.

One proposed mechanism through which the gut microbiota may influence PD pathogenesis includes the initiation of α-synuclein aggregation by bacterial amyloids.^63^ Bacterial amyloids are fibrous proteins^64^ that have been associated with a variety of human diseases including PD.^65^ The gut microbiota has been shown to produce biofilm-associated amyloids that are able to enhance alpha-synuclein aggregation in *C*. *elegans*.^66^ For example, curli is a type of amyloid produced by enteric bacteria^67^ and shown to promote alfa-synuclein aggregation.^68^^,^^69^^,^^70^ Indeed, curli subunits have been found to be present in *E*. *coli* EVs.^45^ EVs secreted by the gut microbiota could transport amyloid proteins as cargo to sites susceptible to a-synuclein aggregation such as the enteric nervous system and the brain, potentially contributing to both body-first and brain-first PD pathogenesis hypotheses.^71^ In this work we did not identify amyloid proteins from our EV proteome data as our proteomics approach was not optimized for detection of low abundancy proteins in the analyzed samples.

PD is associated with intestinal inflammation,^72^^,^^73^^,^^74^ and gut microbiota of patients with PD is able to disrupt intestinal homeostasis leading to inflammation, barrier dysfunction and alpha-synuclein aggregation.^75^ Pathway analysis of gut microbiota of patients with PD showed enrichment in responses to oxidative stress, while depletion was observed in cellular growth, microbial motility and community signaling, potentially indicating responses to inflammatory conditions.^76^ Environmental stress factors can promote EV production and size,^77^^,^^78^^,^^79^ and affect EV protein cargo.^80^^,^^81^ Larger EVs carry greater diversity of proteins compared to their smaller counterparts.^82^ In our study, we observed larger size and quantity of fecal EVs in patients with PD, along with a higher proportion of bacterial proteins compared to healthy controls. It is tempting to speculate that different EV characteristics are related to dysbiotic conditions withing the gut of patients with PD.

Based on 16S rRNA data, several metabolic pathways were depleted in gut microbiota-derived EVs from patients with PD. The most prominent finding within cofactor, carrier and vitamin group concerned menaquinone biosynthesis. Menaquinones (vitamin K2) are membrane-bound electron carriers in bacterial electron transfer chains.^83^ Menaquinones are potential growth factors for other gut bacteria species, and they might be incorporated into EVs.^84^ Menaquinones have been shown to inhibit alpha-synuclein fibrillization^85^ and to exert neuroprotective properties *in vitro*^86^^,^^87^^,^^88^ and in mouse models of neuroinflammation and cognitive decline.^86^ This finding is in line with the observation of lower serum menaquinone levels in patients with PD,^89^ while it contrasts with some previous studies reporting enrichment in predicted pathways related to menaquinone synthesis in the gut microbiota of patients with PD.^74^^,^^76^^,^^90^ Within the secondary metabolite biosynthesis group, we observed lower potential in the biosynthesis of ergothioneine, a microbial antioxidant with protective effects across multiple PD models including fruit fly, *C*. *elegans*, mouse, and dopaminergic cell cultures.^91^ Ergothioneine can both inhibit alpha-synuclein fibrillization and disassemble already formed alpha-synuclein fibrils,^92^ and its serum levels are lower in patients with PD.^93^

We also observed reduced potential for aromatic compound degradation with several pathways related to toluene degradation. Toluene is a neurotoxic organic solvent^94^ which damages dopaminergic neurons in *C*. *elegans*.^95^ Exposure to environmental toxicants has been associated with an increased risk of developing PD.^96^ The gut microbiota plays a dual role in toxicant metabolism, being capable of both degrading and producing harmful compounds.^97^^,^^98^ As the predictive pathway results reflect the metabolic characteristics of the EV producing bacteria, it is worth considering whether the capacity of gut microbiota-derived EVs to support protective functions is reduced in patients with PD, and how this might contribute to the disease pathophysiology. Overall, the pathway analysis points to mechanisms through which gut microbiota-derived EVs could influence PD and warrants direct experimental investigation.

Our study has several strengths. Our study investigates gut microbiota-secreted EVs in the pathogenesis of PD in a controlled clinical setting. Samples were processed individually for 16S rRNA and proteome characterization and the same sample groups were used for both analyses. The presence of isolated EVs was confirmed with TEM, NTA, and western blot. Negative controls were used during analysis in proteomics and 16S rRNA analysis to exclude possible contaminations. Our data are in broad agreement with published results regarding gut microbiota alterations in PD.^10^^,^^13^^,^^51^^,^^52^^,^^53^^,^^54^ With a machine learning approach, we were further able to show dissimilarities in gut microbiota and gut microbiota-derived EVs between patients with PD and healthy controls. This is important because we have recently shown that microbiota-derived EVs represent a distinct entity from the gut microbiota.^99^

In this controlled clinical study, gut microbiota-derived EVs exhibited distinct characteristics in patients with PD compared to healthy controls. These findings suggest that gut microbiota-derived EVs and their molecular cargo are associated with the pathophysiology of PD. Extending the research into the gut microbiota in PD to EVs secreted by the gut microbiota may offer new avenues for diagnostic and therapeutic interventions.

### Limitations of the study

There are several limitations to the present study. Here, we show associations between gut microbiota-derived EVs, isolated from fecal samples, and PD in a controlled clinical study. We did not investigate, however, their possible biological function in PD pathogenesis. The GO-annotation analysis of EV proteins did not allow for the identification of all proteins found. Complete separation of bacterial EVs and host-derived human EVs is not possible with current methodology available even though enrichment of bacterial EVs was performed during the isolation process. Finally, the number of samples was small, which limits the statistical analysis, for instance the effects of sex, diet, or medication factors could not be assessed in this study.

## Resource availability

### Lead contact

Requests for further information and resources should be directed to and will be fulfilled by the lead contact, Sonja Karikka (sonja.karikka@oulu.fi).

### Materials availability

This study did not generate new unique reagents.

### Data and code availability

• The mass spectrometry proteomics data have been deposited with the ProteomeXchange Consortium via the PRIDE^100^ partner repository with the dataset identifier PXD059764 and are publicly available as of the date of publication. This study analyses existing, publicly available 16S rRNA data, accessible at GenBank under the accession number PRJNA1020741 for the control samples^101^ and PRJNA1041975 for the PD samples.^99^ The experimental parameters related to EV isolation and characterization have been submitted to the EV-TRACK knowledgebase^102^ with the EV-TRACK ID: EV250042 and are publicly available as of the date of publication.
• All original code has been deposited at Zenodo and is publicly available at https://doi.org/10.5281/zenodo.15101267 as of the date of publication.
• Any additional information required to reanalyze the data reported in this study is available from the lead contact upon request.

## Acknowledgments

Mass spectrometry analysis was performed at the Turku Proteomics Facility, University of Turku and Åbo Akademi University. The facility is supported by 10.13039/501100013840Biocenter Finland. The authors thank Pirkko Holappa for her help in recruiting study participants at Oulu University Hospital. This work was supported by the 10.13039/501100006432University of Oulu Graduate School, 10.13039/100030905Finnish Parkinson Foundation, Oulu Medical Research Foundation, the Finnish Brain Foundation (to S.K.), and the 10.13039/501100004212Päivikki and Sakari Sohlberg Foundation (to M.V.T.). Funders played no role in the study design, data collection, analysis and interpretation of data, or writing of this manuscript.

## Author contributions

A.S., J.R., and T.R.-L. supervised and conceptualized the project. S.K., S.M., J.S., J.K., and A.M.P. arranged the sample collection. S.K., J.H., N.V., S.M., A.Z., N.S., M.S., S.J.V., L.L., and P.P.E. took part in the methodology design and experimental work. S.K., A.K., J.H., J.T., and M.V.T. performed the data analysis. S.K., M.V.T., J.T., A.S., J.R., and T.R.-L. wrote the original draft. All authors read and approved the final manuscript.

## Declaration of interests

The authors declare no competing interests.

## STAR★Methods

### Key resources table

**Table:** 

REAGENT or RESOURCE	SOURCE	IDENTIFIER
**Antibodies**		

Goat polyclonal anti-rabbit IgG	Agilent Technologies	Cat#P0488; RRID: AB_3718618
Rabbit polyclonal anti-CD63	Santa Cruz	Cat#sc-15363; RRID: AB_648179
Rabbit polyclonal anti-OmpA	ARC	Cat#111228

**Biological samples**		

Fecal samples	Oulu University Hospital, Oulu, Finland	N/A

**Chemicals, peptides, and recombinant proteins**		

Invitrogen™ SYPRO™ Ruby protein gel stain	Thermo Fisher Scientific	Cat#S12000
Lumi-Light Western Blotting Substrate	Roche Diagnostics	Cat# 12015200001
OptiPrep™ (iodixanol 60% w/v)	STEMCELL Technologies	Cat# 07820
Phusion Flash High-Fidelity PCR Master Mix	Thermo Fisher Scientific	Cat#F548L

**Critical commercial assays**		

BCA Protein Assay Kit	Pierce	Cat#23277
Quant-iT PicoGreen dsDNA Assay Kit	Thermo fisher Scientific	Cat# P11496

**Deposited data**		

16S rRNA sequencing data (Healthy controls)	Mishra et al.^101^	GenBank: PRJNA1020741
16S rRNA sequencing data (patients with PD)	Kaisanlahti et al.^99^	GenBank: PRJNA1041975
Experimental parameters of extracellular vesicle isolation and characterization	This paper	EV-TRACK: EV250042
Proteomics raw data	This paper	PRIDE: PXD059764

**Oligonucleotides**		

16S rRNA specific primer S-∗-Univ-0515-a-S-19	This paper	5′-GTGCCAGCMGCCGCGGTAA-3′
S-∗-Univ-0519-a-S-18	This paper	5′-AGCMGCCCGCGGTAATWC-3′
S-D-Bact-0907-a-A-20	This paper	5′-CCGTCAATTCCTTTRAGTTT-3′

**Software and algorithms**		

Generated code	This paper	https://doi.org/10.5281/zenodo.15101267
GraphPad Prism Ver. 10.1.2	GraphPad Software	N/A
Peaks Studio Ver. 10.6	Bioinformatics Solutions	N/A
QIIME2 Ver. 2022.2; 2023.5; 2024.5-amplicon	Bolyen et al.^103^	https://qiime2.org/
R Ver. 4.3.1; 4.5.2	R Core Team	https://www.r-project.org/
RStudio Ver. 2023.03.0 + 386; 2025.05.0 + 496	Posit Software	https://posit.co/products/open-source/rstudio
Xcalibur Ver. 4.1	Thermo Fisher Scientific	N/A

**Other**		

Amicon® Ultra-15 Centrifugal filter units (100 kDa)	Merck	Cat# UFC910024
AMPure XP	Beckman Coulter	Cat# A63881
Easy nLC 1200	Thermo Fisher Scientific	N/A
exoRNeasy Midi Kit	Qiagen	Cat# 77144
Exo-spin™ mini-columns	Cell Guidance Systems	Cat#EX03-50
Falcon® Cell Strainer filter	Corning	Cat#352340
Ion Torrent PGM Sequencer	Thermo Fisher Scientific	N/A
iScript cDNA synthesis kit	Bio-Rad Laboratories AB	Cat#1708891
Nalgene™ Rapid-Flow™ Sterile Disposable Filter Units with PES membrane (150 mL)	Thermo Fisher Scientific	Cat#165-0045
Nalgene™ Rapid-Flow™ Sterile Disposable Filter Units with PES membrane (1000 mL)	Thermo Fisher Scientific	Cat#167-0045
Q Excative HF Mass Spectrometer	Thermo Fisher Scientific	N/A
QIAamp Fast DNA Stool Mini Kit	Qiagen	Cat# 51604

### Experimental model and study participant details

Ethical approval for the study was granted by the regional medical research ethics committee of the Wellbeing services county of North Ostrobothnia (EETTMK:11/2019). Healthy controls and patients with PD provided informed consent before participating in the study. Fecal samples were collected from 20 patients diagnosed with PD (9 females, 11 males, mean age 69 years, range 63–73) and followed up at the neurological outpatient clinic of the Oulu University Hospital and 19 healthy controls (10 females, 9 males, mean age 65 years, range 40–86). Time between PD diagnosis and sample collection was 7.7 ± 7 years (range 0–22). All patients with PD were receiving dopaminergic antiparkinsonian medication. Healthy controls were defined as individuals with no neurodegenerative diseases or observable gastrointestinal issues based on colonoscopy. Among the study participants, 95% of healthy controls and all patients with PD were of Finnish ethnicity. Additional clinical and demographic details are provided in Table S1. Patients were provided with a sample collection kit which they used for sample collection and submission. Samples were sent at ambient temperature. Latency between initial sample collection and arrival was monitored. Samples that took more than 3 days to arrive were discarded. Samples were stored upon arrival at −80°C. None of the participants reported any inflammatory bowel diseases, use of tobacco products, or antibiotic administration in the three months prior to sample collection.

### Method details

#### Isolation of gut microbiota-derived extracellular vesicles from fecal samples

Isolation was done in line with the previous publication^104^ and current recommendations,^32^^,^^105^ and experimental parameters were submitted to the EV-TRACK knowledgebase (EV-TRACK ID: EV250042).^102^ 1000 mg of thawed ice-cold fecal material was suspended in 15 mL of ice-cold 1*x* sterile-filtered Dulbecco’s phosphate buffered saline (DPBS) (Sigma-Aldrich, USA). The DPBS was sterile-filtered prior to use with 0.45 μm Nalgene Rapid-Flow Sterile Disposable Filter Units with PES membrane (Thermo Fisher Scientific, 1000 mL, USA). The fecal suspension was centrifuged twice at 10,000 g for 30 min at 4°C. The supernatant was filtered with a 40 μm Falcon® Cell Strainer filter (Corning, USA) and sterile-filtered using 0.45 μm Nalgene™ Rapid-Flow™ Sterile Disposable Filter Units with PES membrane (Thermo Fisher Scientific, 150 mL, USA) to remove cell debris and other particles. The supernatant was concentrated to 0.2–1 mL using Amicon® Ultra-15 Centrifugal filter units with a 100,000 Da molecular weight cut-off (Merck, USA) according to the manufacturer’s instructions. The EVs were purified using size-exclusion chromatography with Exo-spin mini-columns (Cell Guidance Systems, UK) according to the Exo-Spin Exosome Purification Kit instructions. 200 μL of fecal concentrate was introduced for purification per individual sample. The EVs were further purified with density gradient ultracentrifugation by creating 40%, 20%, 10%, and 5% iodixanol gradients using OptiPrep™ (STEMCELL Technologies, Canada). 200 μL of sample was administered on top of the 5% gradient and ultracentrifuged at 100,000 *x* g for 18h at 4 C°. Fractions 6 and 7 contained the majority of bacterial EVs,^104^ and thus were collected for washing. The bacterial EV-containing fractions were washed by mixing 1 mL of the fractions with 9 mL of DPBS. The EVs were pelleted by ultracentrifugation at 100,000 *x* g for 2.5h at 4 C°. The supernatant was discarded and the EVs were suspended in 100 μL of DPBS and stored at −80 C°. Sterile DPBS was used as a negative control that was carried over to EV isolation and further procedures to exclude possible contaminants at the analysis phase.

#### Protein isolation from extracellular vesicles

Proteins were individually isolated from each EV sample. The EVs from fractions 6 and 7 were combined and 100 μL of EV sample was used for protein isolation. The EVs were suspended in 300 μL of water, 400 μL of ethanol, and 100 μL of chloroform. Samples were vortexed thoroughly and centrifuged at 14,000g for 1 min. The water-methanol interface was removed. 400 μL of methanol was added to the samples. The samples were vortexed thoroughly and centrifuged at 16,000 g for 5 min. The supernatant was removed and the samples were air-dried at room temperature until they were completely dry (approximately 20 min). 5 μL of 4*x* Laemmli sample buffer (Bio-Rad Laboratories AB, USA) and 15 μL of water were added to the samples. The samples were heated for 5 min at 95°C and loaded on Mini-Protean TGX 12% separating gels (Bio-Rad Laboratories AB, USA). The gels were run at 110 V for 10–15 min, until the Laemmli buffer migrated 1 cm into the gel. The gels were fixed in 50% ethanol and 10% acetic acid solution in a shaker for 30 min. The gels were rinsed with water and stained overnight with Invitrogen™ SYPRO™ Ruby protein gel stain (Thermo Fisher Scientific, USA). The gels were de-stained with 5% acetic acid in a shaker for 5 min and washed in water for 15 min in a shaker. Protein bands were cut from the gel under UV-light and dehydrated with absolute ethanol.

#### Mass spectrometry

The dried gel-incorporated EV protein samples were analyzed at the Turku Bioscience proteomics facility. The gels were rehydrated and washed with water. The proteins were reduced with mM 1,4-dithiotreitol for 30 min following dehydration of the gels with 100% acetonitrile (ACN). The gels were alkylated with 55 mM iodoacetamide for 20 min in the dark at room temperature and washed twice with 100 mM ammonium bicarbonate. The gels were dehydrated with ACN and dried with vacuum centrifugation for 5 min. The gels were digested with 0.02 μg/μL trypsin for 20 min and covered with 10% ammonium bicarbonate solution overnight at 37°C. Peptide extraction was performed by treating the gels with 100% ACN. The gels were vortexed and incubated for 15 min at 37°C. The supernatant was collected. Peptide extraction was repeated with 50% ACN/5% formic acid solution. The supernatant was dried with vacuum centrifugation and the samples were stored at −20°C. For mass spectrometry, the samples were suspended in formic acid. Liquid chromatography-electrospray ionisation-tandem mass spectrometry (LC-ESI-MS/MS) analysis was performed with a nanoflow HPLC system (Easy-nLC1200, Thermo Fisher Scientific, USA) coupled with a Q Exactive HF mass spectrometer (Thermo Fisher Scientific, USA) equipped with a nano-electrospray ionisation source. The samples were loaded on a trapping column and separated on a 15 cm C18 column (75 μm × 15 cm, ReproSil-Pur 5 μm 200 Å C18-AQ, Dr. Maisch HPLC GmbH). The mobile phase included water with 0.1% formic acid (solvent A) and ACN/water (80:20 v/v) with 0.1% formic acid (solvent B). Peptides were eluted with linear gradient (8%–43%) of solution B for 10 min. Data was received using Thermo Xcalibur 4.1 software (Thermo Fisher Scientific, USA). A mass range of 300–2000 m/z was covered including high-energy collisional dissociation fragmentation of the 10 most intense peptide ions.

Data analysis of the proteomic data was performed using PEAKS Studio software (version 10.6, Bioinformatics Solutions Inc.). The proteomic data was searched against the UniProt Swissprot and UniProt trEMBL databases (UniProt database version 2022_05). Error tolerance was adjusted to 10 ppm and 0.02 Da for parent mass and fragment mass tolerance, respectively. The false discovery rate was set to 1% for peptides and proteins. A coverage threshold of 1% was set for accepted protein identification. The results were processed with RStudio (Version 2023.03.0 + 386) and the figures were drawn with the ggplot2 package (version 3.5.1). Sterile DPBS, treated the same way as the samples, was used as a negative control to exclude the possibility of contaminants in the samples. Proteins with different IDs were treated as unique proteins. Overall, 5,159 peptides and 1,109 proteins were identified using the UniProt Swissprot database, and 18,674 peptides and 12,353 proteins were found using the Uniprot TrEMBL database. Available GO-annotations were retrieved from the UniProt database and proteins with similar ancestry were grouped together.

#### Nanoparticle tracking analysis

Nanoparticle tracking analysis was conducted to determine the concentration and size distribution of particles in fecal EV samples. Measurements were taken using a Nanosight NM300 (Malvern, UK) with Nanosight NTA software (version 3.4.4). EV samples were diluted 1:50 or 1:100 prior to measurement. One measurement included 4 × 60 s captures and the results from each repetition were merged to obtain mean values for particle size and concentration. The results were analyzed with GraphPad Prism (version 10.1.2). After testing for normality using D’Agostino & Pearson, Anderson-Darling, Shapiro-Wilk, and Kolmogorov-Smirnov tests, the significance of size and concentration of particles in patients with healthy controls vs. patients with PD was calculated using the parametric unpaired *t* test (size distribution) and unpaired *t* test with Welch correction for unequal variances (concentration).

#### Transmission electron microscopy

The purity and presence of EVs was confirmed with transmission electron microscopy (TEM). EVs were negatively stained at the Biocenter Oulu Electron Microscopy Core Facility. Briefly, 5 μL of EVs were introduced to a Formvar-carbon coated and glow-discharged copper grid (400 mesh) and incubated for 20 min. Excess sample was removed and the grid was rinsed a few times in a droplet of water. The grid was fixed in 1% glutaraldehyde for 5 min and rinsed for 8 × 1 minutes with water. The grid was stained with a droplet of neutral 2% uranyl acetate. The grid was washed and coated with 2% methylcellulose-uranyl acetate for 10 min. Excess fluid was removed from the grid with Whatman no. 1 filter paper and it was air-dried before imaging. The stained EVs were imaged with a Tecnai G2 Spirit 120 kV transmission electron microscope (FEI Europe) with Veleta and Quemesa CCD cameras.

#### Western blot

Western blot analysis was used to assess the presence of bacterial and human EVs. The EV protein concentration was measured via BCA Protein Assay Kit (23227, Pierce). 2 μg of EV proteins were mixed with 4*x* Laemmli loading buffer (J60015-AD, Thermo Scientific) and boiled at 95°C for 5 min. Proteins were concentrated on 4% SDS PAGE gel and separated on 12% SDS PAGE gel, and then transferred to nitrocellulose membrane (741280, Macherey-Nagel). After being blocked for 30 min, membranes were probed at +4°C overnight with the following antibodies: CD63 (1:1000, sc-15363, Santa Cruz) and OmpA (1:1000, 111228, ARC) in 5% milk. The secondary peroxidase conjugated IgG antibodies (1:5000, P0448, Agilent Technologies) were applied to the membranes. The Lumi-Light Western Blotting Substrate (Roche Diagnostics) was used to visualize signal. PageRuler Plus Prestained Protein Ladder (26616, Thermo Scientific) was used as the molecular weight marker.^104^

#### DNA isolation from fecal samples

For gut microbiota analysis a total fecal DNA was isolated using a QIAamp Fast DNA Stool Mini Kit (Qiagen, Germany) according to the manufacturer’s instructions. Briefly, 200 mg of fecal material per sample was suspended in InhibitEX buffer followed by lysis and binding of the DNA to the silica membrane of QIAmp Mini spin columns. Contaminants were removed by washing and the DNA was eluted in 200 μL of ATE buffer and stored at −20°C for further use.

#### RNA isolation from fecal extracellular vesicles

For 16S rRNA analysis of gut microbiota-derived EVs, a total RNA was isolated from individual fecal EV samples using an exoRNeasy Midi Kit (Qiagen, Germany). We chose to use RNA isolation since RNA is actively packed into EVs,^106^ while DNA in EVs may be a result of cell lysis.^107^ Isolation was performed according to the manufacturer’s instructions. Briefly, 10 μL of gut microbiota-derived EVs were suspended in 90 μL DPBS followed by lysis with QIAzol. Chloroform was added to the suspension. The upper phase was collected and mixed with ethanol, which was then administered to RNeasy MinElute spin columns to bind the RNA. The columns were washed and the RNA was eluted with nuclease-free water. The isolated RNA was stored at −80°C for further use.

#### Conversion of RNA to cDNA

The RNA samples were converted to cDNA with an iScript™ cDNA synthesis kit (Bio-Rad Laboratories AB, USA) according to the manufacturer’s instructions. Approximately 200 ng of extracted RNA and 0.5 μM 16S rRNA specific primer S-∗-Univ-0515-a-S-19 (5′-GTGCCAGCMGCCGCGGTAA-3′) were used in a total volume of 20 μL.

#### Polymerase chain reaction (PCR) amplification and 16S rRNA gene sequencing

The V4–V5 hypervariable region of the 16S gene of EV cDNA and fecal DNA were amplified using the S-∗-Univ-0519-a-S-18 (5′-CAGCMGCCCGCGGTAATWC-3′) and S-D-Bact-0907-a-A-20 (5′-CCGTCAATTCCTTTRAGTTT-3′) primers. To enable multiplexing, the forward primer contained a unique 9 bp barcode. Amplification was carried out in duplicate 15-μL PCR reactions following the manufacturer’s protocol, utilising Phusion Flash High Fidelity PCR master mix (Thermo Fisher Scientific, USA), 10 ng of fecal DNA or 4 μL of cDNA, and 0.5 μM of forward and reverse primers. The sequencing run included two negative controls and two mock community controls (HM-782D and Microbial Mock Community B). Amplification was conducted using a Biosystems Veriti 96-Well Thermal Cycler (Thermo Fisher Scientific, USA) with an initialisation phase of 3 min at 98°C, 22 cycles of amplification with an annealing temperature of 64°C, and a final elongation phase of 5 min at 72°C.

Following amplification, duplicate reactions were combined, and the presence of PCR products was confirmed through agarose gel electrophoresis. For sequencing, PCR products were purified using AMPure XP (Beckman Coulter, CA, USA) reagent, and its concentrations were measured with Bioanalyser DNA-1000 chips. Equivalent amounts of products for each sample were pooled together. Multiplexed samples underwent further purification using AMPure XP reagent and were analyzed with Bioanalyser DNA-1000 chips; final DNA concentrations were measured with a Quant-iT PicoGreen dsDNA Assay Kit (Thermo Fisher Scientific, USA). Sequencing was performed with an Ion Torrent PGM sequencer using an Ion PGM Hi-Q View template kit (400 bp templating program), an Ion PGM Hi-Q View sequencing kit (850 cycles), and a 316 v2 chip.

#### 16S rRNA sequence analysis

Analysis of the 16S rRNA sequence was conducted using Quantitative Insights into Microbial Ecology 2 (QIIME2; version 2022.2).^103^ Reads shorter than 200 bp were excluded. The selected reads underwent demultiplexing and denoising using the divisive amplicon denoising algorithm 2 (DADA2)^108^ implemented in QIIME2, with removal of chimeric reads. Following this, reads were trimmed at base 30 and truncated at base 330, guided by the quality assessment plots generated by QIIME2 during demultiplexing. Bacterial taxa were assigned to representative sequences using the SILVA database (version 138.1).^109^ Taxa such as mitochondria, eukaryota, cyanobacteria, and archaea were excluded from further analysis. Contaminant reads were filtered out using negative controls and the R decontam package (version 1.8.0) with a prevalence-based method and a threshold of 0.5.^110^ The processed data had mean frequencies of 12,377 and 11,205 and median frequencies of 11,492 and 9,634 in the EVs and fecal samples, respectively. Reads were rarefied to a sampling depth of 4,707 for EVs and 4,330 for fecal samples. The lowest read count prior to filtering was 728 for EV samples. After rarefying to 4,707 reads, one EV sample from healthy controls was excluded.

Alpha diversity, assessing within-sample diversity, was estimated using the number of observed features, Chao1 index, and the Shannon index, which are commonly used measures of alpha diversity in microbiome research. The number of observed features captures sample richness, Chao1 emphasizes low-abundance taxa, and the Shannon index incorporates both richness and evenness.^111^ Thus, these indices have been applied together in studies on bacterial EVs.^112^^,^^113^^,^^114^ In this study, we used all three indices to provide a more comprehensive assessment of the alpha diversity of gut microbiota and gut microbiota-derived EVs, and to minimize potential bias associated with relying on a single metric.^115^ The statistical significance of alpha diversity differences was determined using the Kruskal-Wallis H test, with a significance threshold of *p* ≤ 0.05. *p* values were adjusted with Benjamini-Hochberg correction. Beta diversity, evaluating between-sample diversity, was assessed using principal coordinate analysis (PCoA) with the Bray-Curtis dissimilarity index. Rarefaction was performed for data normalisation based on total read counts, with the lowest read count setting the threshold. Statistical significance of beta diversity results was tested using PERMANOVA.^116^ Visualisation of alpha diversity was carried out in RStudio (version 2023.03.0 + 386) using the microeco package (version 0.15.0).^117^ PCoA figures using Bray-Curtis Dissimilarity were created in the R package mia (version 1.18.0). The relative abundance data were agglomerated to genus level before analysis.

#### Differential abundance analysis

We used Analysis of Compositions of Microbiomes with Bias Correction (ANCOM-BC)^118^ to analyze differential abundance between healthy controls and patients with PD. P-values less than 0.05 were considered significant and Bonferroni correction was used as a method of adjusting the *p*-values. Analysis was performed on QIIME2 (version 2023.5).^103^ Figures were generated using the R (version 4.3.1) packages pROC (version 1.18.5) and ggplot2 (version 3.4.4).

#### Sample classification using machine learning

To further examine the taxonomic signatures of patients with PD and healthy controls, we trained a classifier using a Random Forest (RF) nested cross-validation algorithm, where different iterations of the training are performed so that each sample is used in both a training and a test set. We used a random state of 123 and 150 estimators. The number of cross-validations was set to 5, and the average of the cross-validations was reported as a result. The ratio of samples in the training set and the test set during the classifier training was 80/20.

#### PICRUSt2 analysis

We predicted metabolic pathways for fecal and EV samples using Phylogenetic Investigation of Communities by Reconstruction of Unobserved States 2 (PICRUSt2).^119^ For the analysis, we used q2-picrust2 plugin available for QIIME2 version 2024.5-amplicon. We utilized epa-ng^120^ and gappa^121^ for the phylogenetic placement of reads, castor^122^ for hidden state prediction, and MinPath^123^ for pathway interference.

The results were visualized in R (version 4.5.2) with ggpicrust2 (version 2.5.6).^124^ In short, metabolic pathway abundances were compared between patients with PD and control samples, using the differential abundance analysis method LinDA^125^ and false discovery rate (FDR) adjustment for *p*-values. Pathways were annotated and manually grouped using the MetaCyc database.^126^ The figure was generated with RStudio (version 2025.05.0 + 496) using ggplot2 (version 4.0.2).

### Quantification and statistical analysis

Descriptions of quantification and statistical analyses are included in the method details and figure legends. Statistical tests used in this study include the Kruskal-Wallis H test with Benjamini-Hochberg correction, PERMANOVA, ANCOM-BC with Bonferroni correction, LinDA with FDR adjustment, unpaired parametric t-tests, and unpaired t-tests with Welch correction. Analyses were performed using R, RStudio, QIIME2, and GraphPad Prism.

## Supplemental information

Document S1. Figures S1–S6 and Tables S1–S5

Data S1. Full list of differentially abundant predicted metabolic pathways in gut microbiota-derived EV samples, related to Figure 7
