# Supplementary material for: Gut microbiota-derived extracellular vesicles in Parkinson’s disease
Source: iScience. 2026 Aug 5;29(8):117057. doi: 10.1016/j.isci.2026.117057 (PMC13474086; doi:10.1016/j.isci.2026.117057)
Supplement: Supplementary file 1 [file mmc1.pdf]

iScience, Volume *29*

Supplemental information

Gut microbiota-derived extracellular vesicles in Parkinson's disease

Sonja Karikka, Mysore V. Tejesvi, Jenni Hekkala, Jenni Turunen, Anna Kaisanlahti, Nikke Virtanen, Surbhi Mishra, Artem Zhyvolozhnyi, Nelli Sinkkonen, Marko Suokas, Sanna Meriläinen, Pande Putu Erawijantari, Leo Lahti, Juha Saarnio, Johanna Krüger, Anne M. Portaankorva, Seppo J. Vainio, Terhi Ruuska-Loewald, Justus Reunanen, and Anatoliy Samoylenko

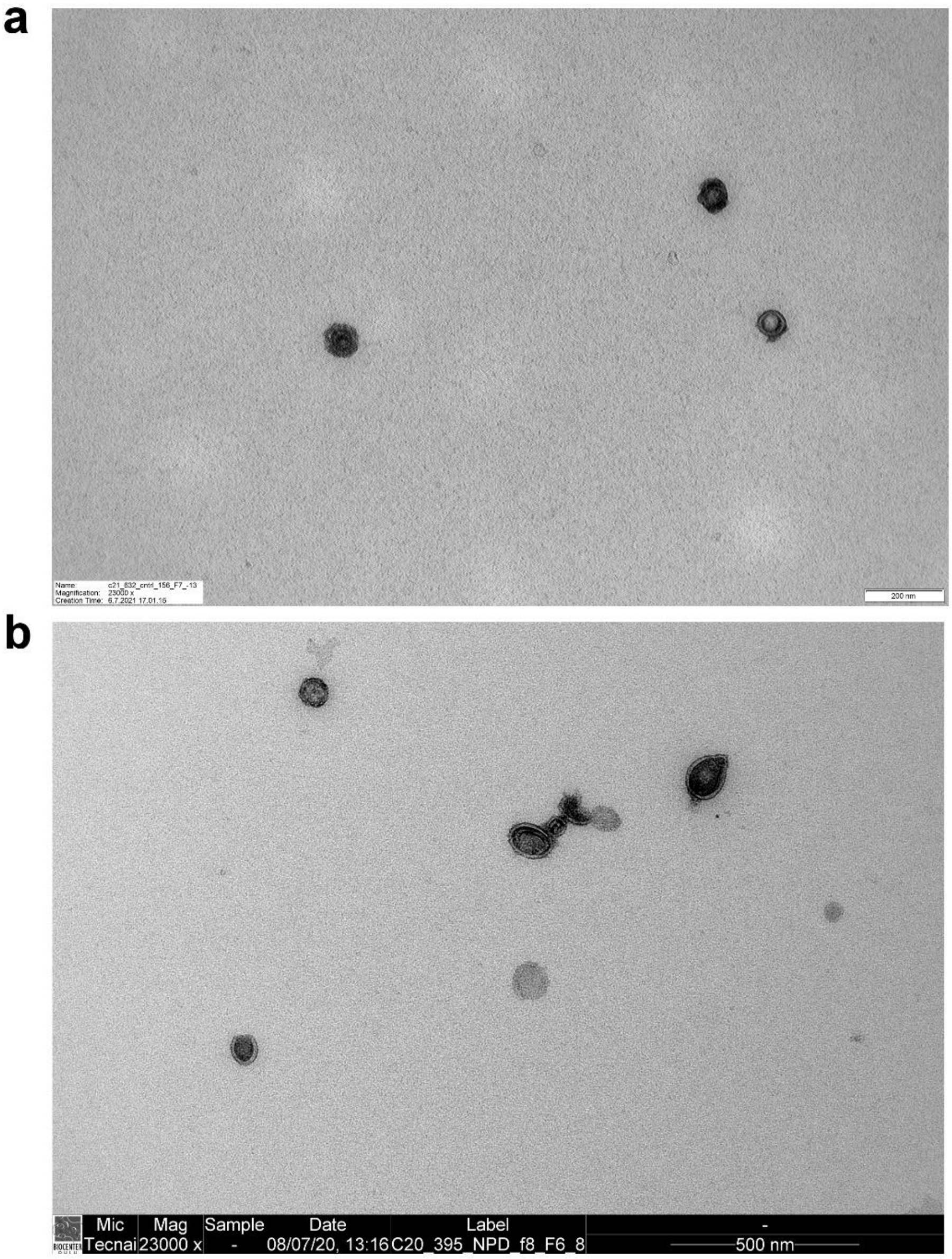

Figure S1. Uncropped transmission electron microscopy images (23,000 X magnification) of gut microbiota-derived extracellular vesicles. Related to Figure 1. A. Healthy controls. **B.** Patients with Parkinson's disease (PD).

a b

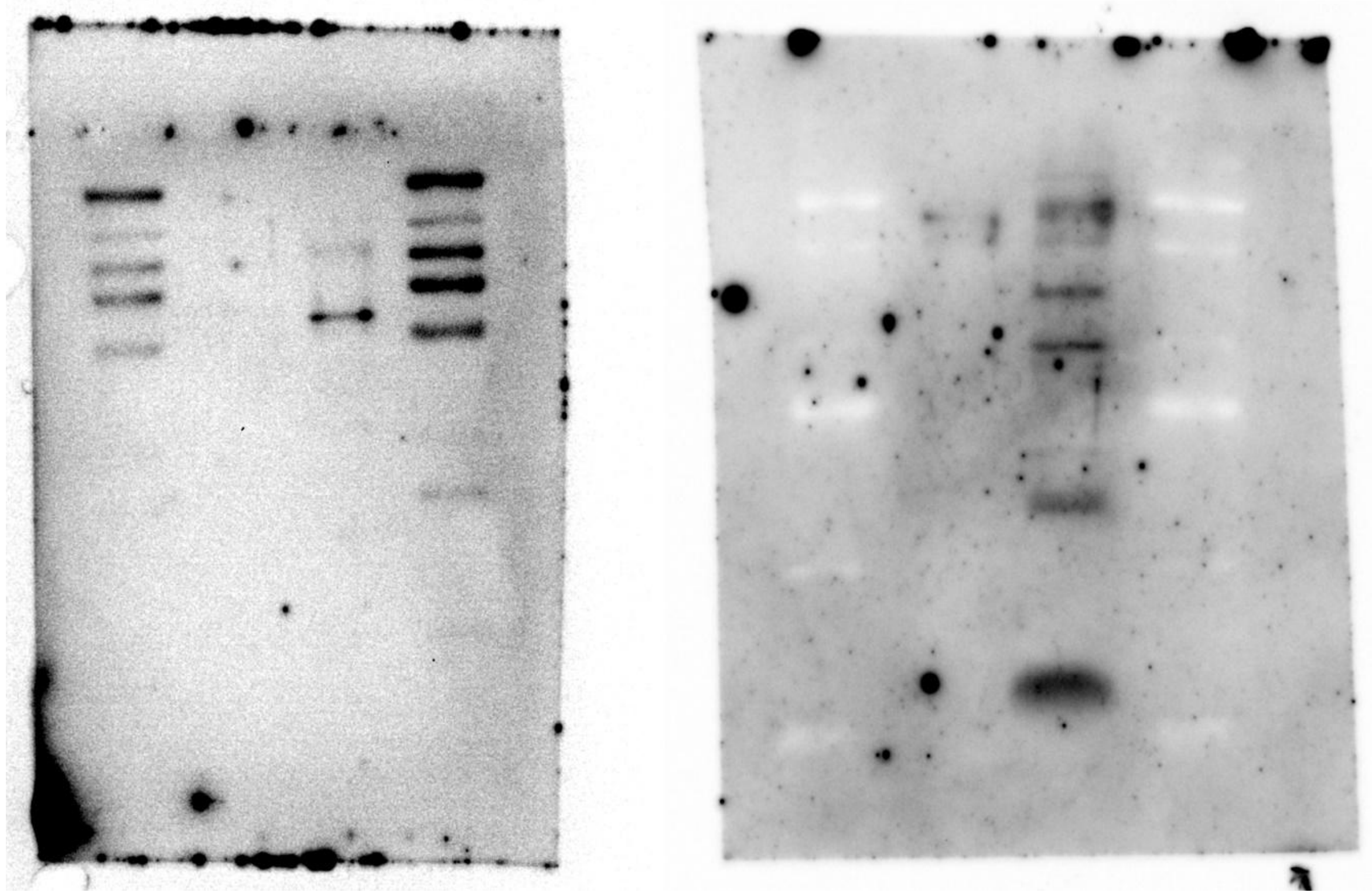

Figure S2. Uncropped blot images. Related to figure 1. A. CD63. **B.** OmpA.

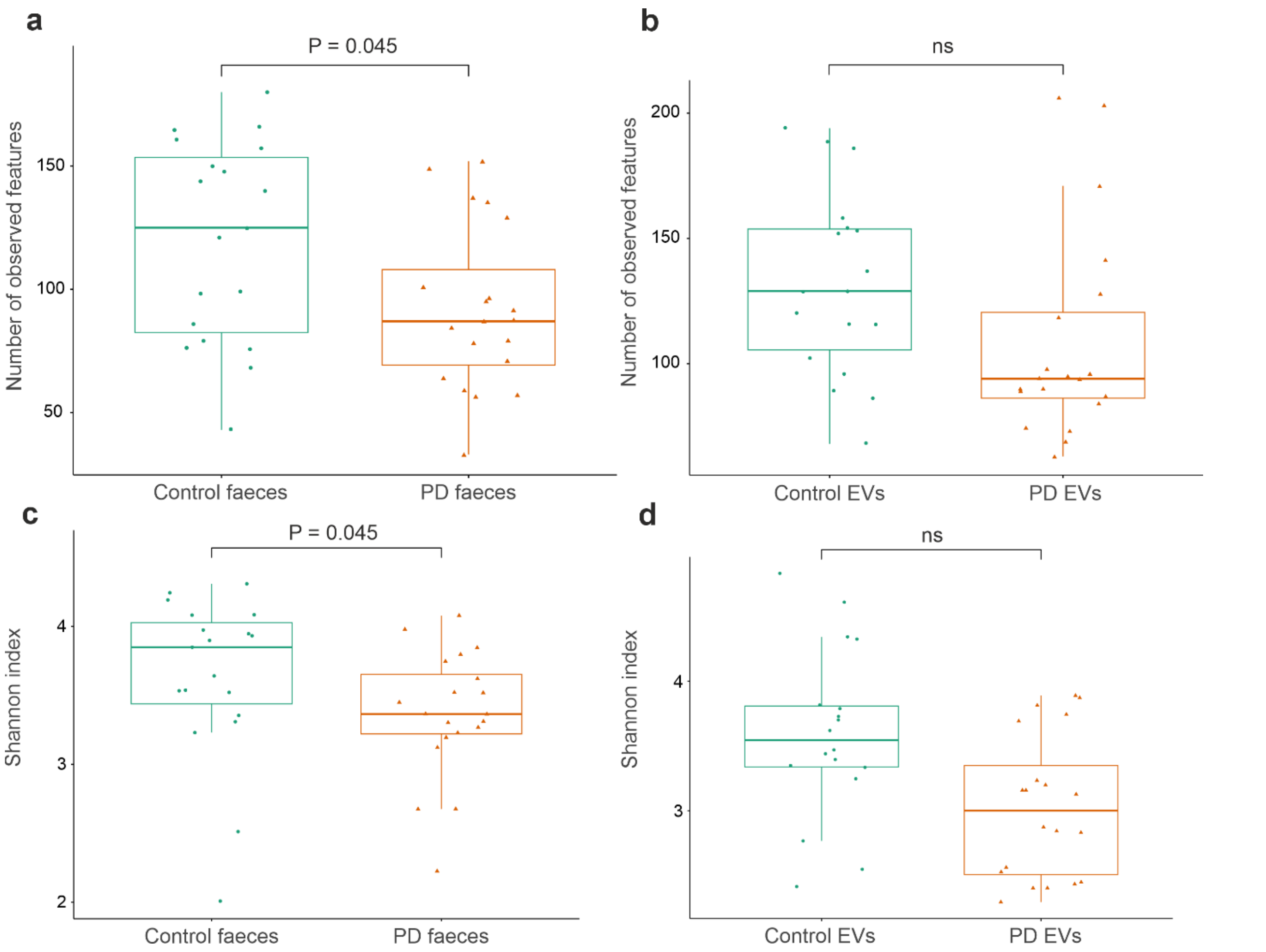

Figure S3. Alpha diversity of gut microbiota and gut-microbiota-derived extracellular vesicles (EVs). Related to Figure 2. A, B. Number of observed features. **C, D**. Shannon index. Significance was determined using the Kruskal–Wallis H test and P-values were adjusted with Benjamini-Hochberg correction. ns: not significant. PD: Parkinson's disease.

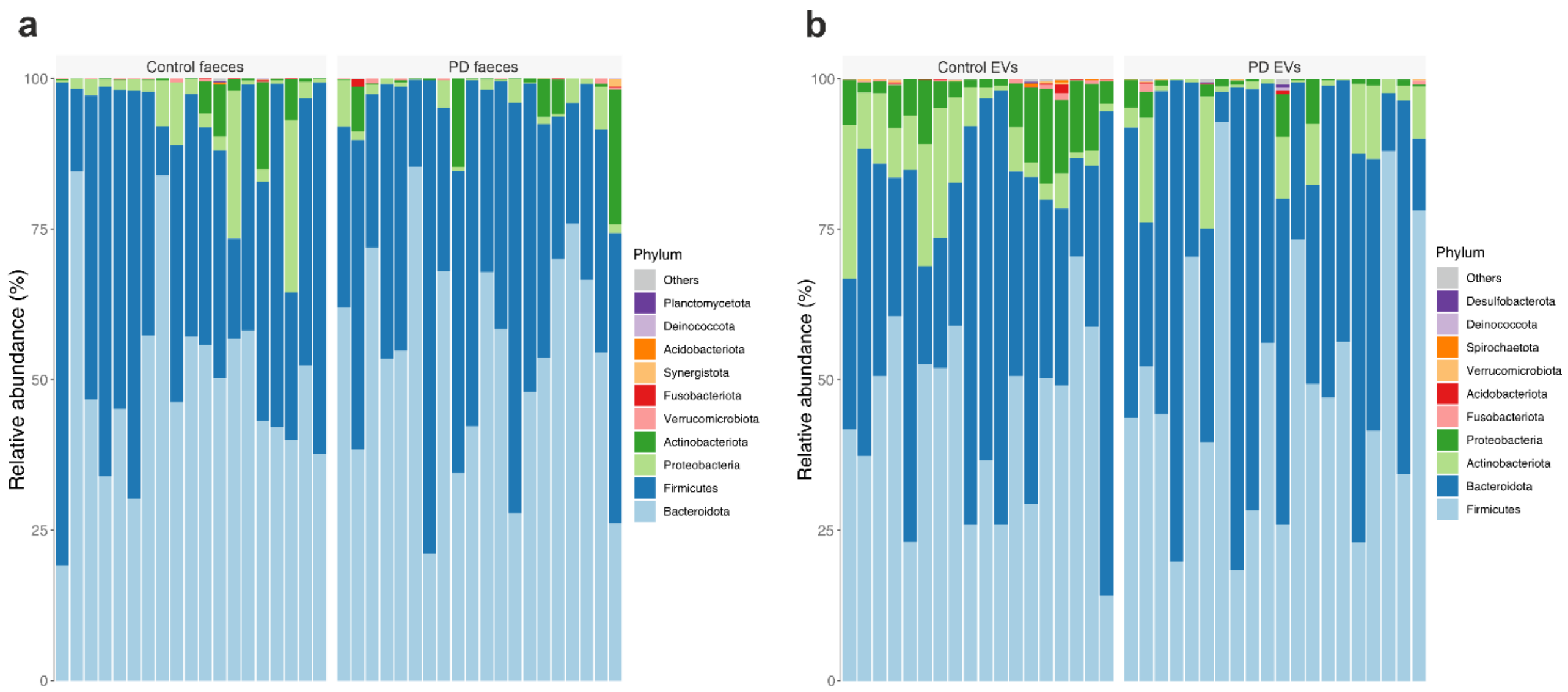

Figure S4. Relative abundance in individual healthy controls and patients with Parkinson's disease (PD) at phyla level. Related to Figure 4. A. Gut microbiota composition **B.** Taxonomic composition of extracellular vesicle (EV) origin in faeces.

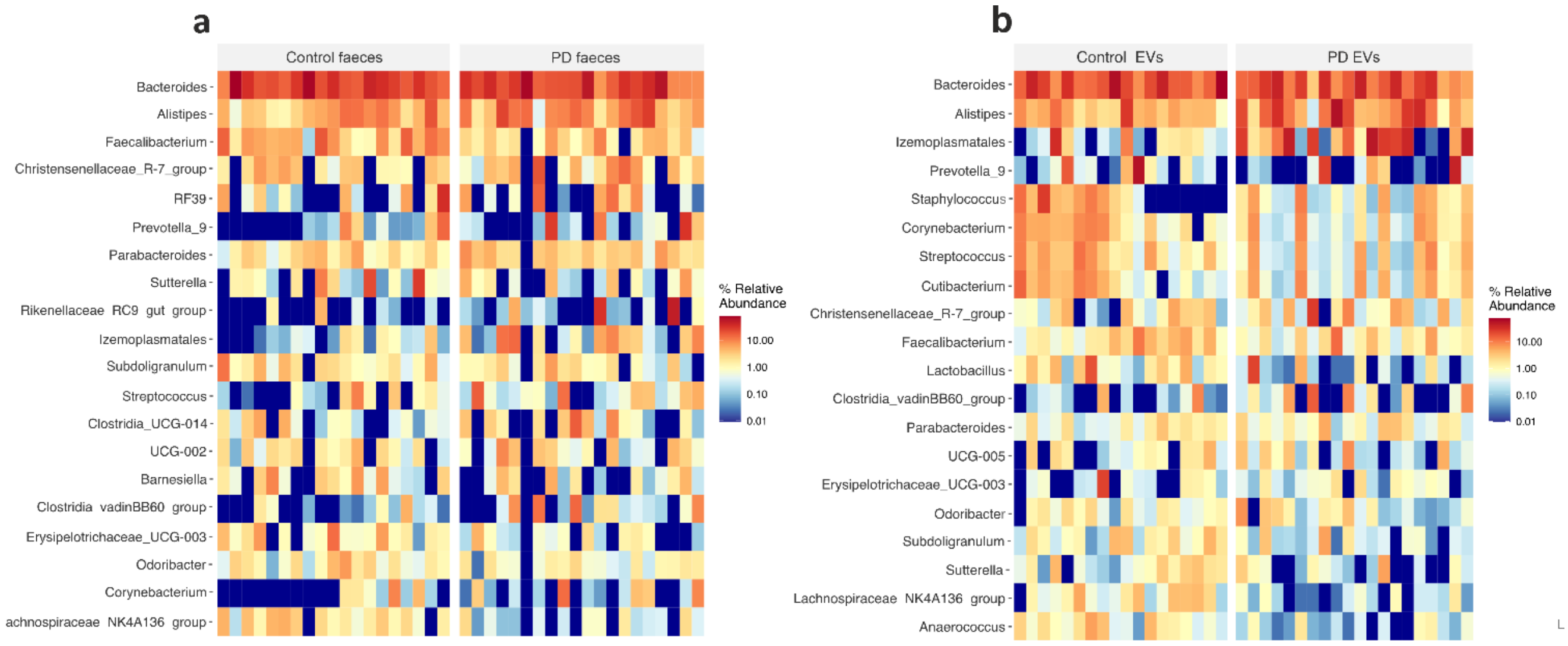

Figure S5. Relative abundance in individual healthy controls and patients with Parkinson's disease (PD) at genera level. Related to Figure 4. A. Gut microbiota composition **B**. Taxonomic composition of extracellular vesicle (EV) origin in faeces.

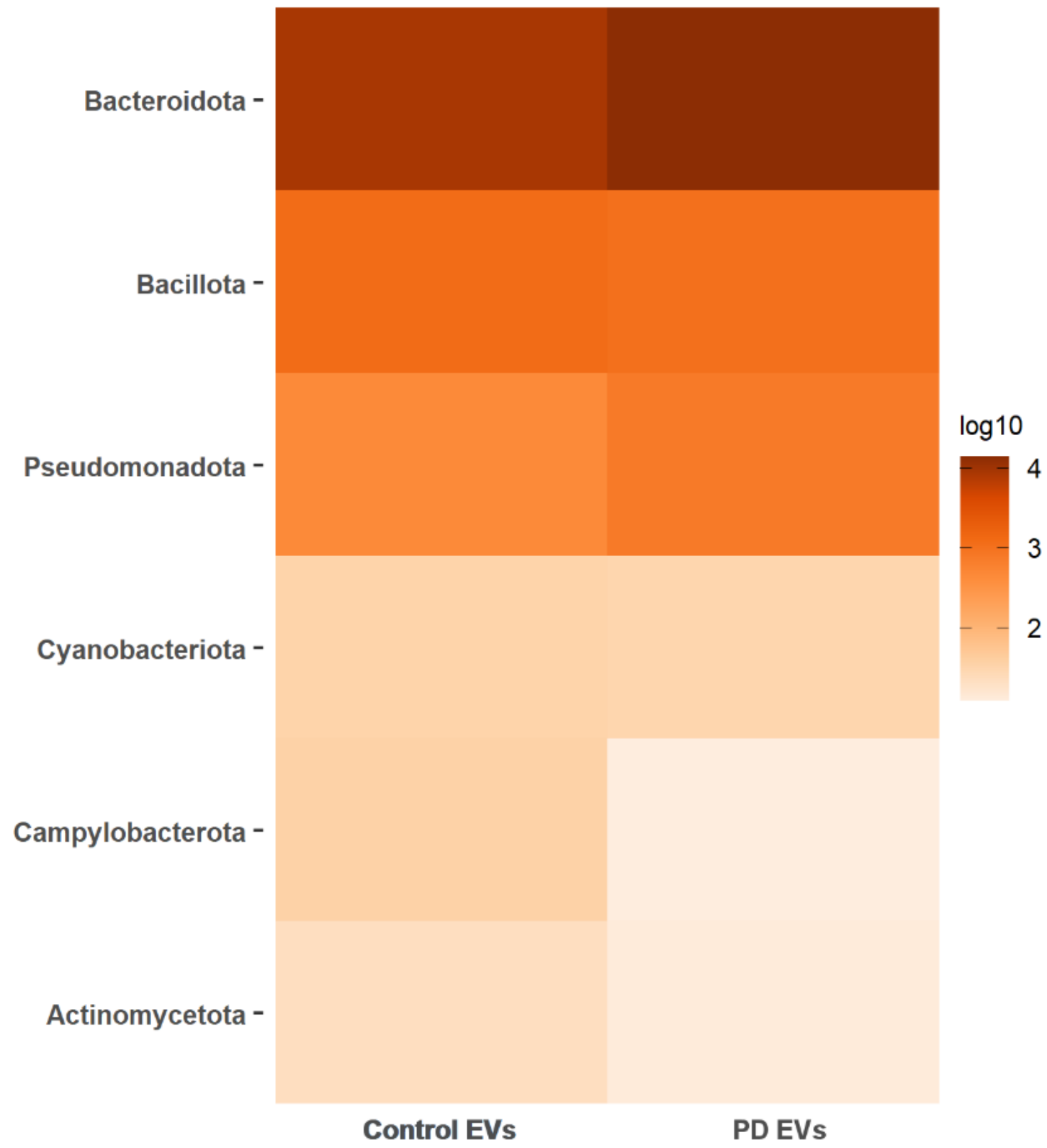

Figure S6. Pooled heatmap showing the most prominent bacterial phyla associated with observed protein hits. Related to Figure 8. Figure shows proteins present in gut microbiota-derived extracellular vesicles (EVs) of healthy controls and patients with Parkinson's disease (PD) with base-10 logarithmic scaling. The phylum names were obtained from the UniProt database, which uses updated phylum nomenclature. The updated phyla names and synonyms are Bacteroidota (Bacteroidetes), Bacillota (Firmicutes), Pseudomonadota (Proteobacteria), Cyanobacteriota (Cyanobacteria), and Actinomycetota (Actinobacteria).

Table S1. Clinical and demographic characteristics of the study population. Related to STAR methods.

**Characteristic**	Control subjects (n = 19)	Patients with PD (n = 20)
Age (SD)	65 (11)	69 (3)
Sex (females)	10 (53%)	9 (45%)
Ethnicity (%)	Finnish (95%); n/a (5%)	Finnish (100%)
BMI (SD)	27.7 (3.8)	27.8 (3.3)
Antibiotic use prior 3 months	0	0
Tobacco smoking	0	0
**Background illnesses**	**Subjects affected**	**Subjects affected**
Asthma	4	1
Cardiovascular diseases	12	11
Endocrinological diseases	4	4
Ophthalmological diseases	0	2
Other neurological diseases	0	4
Sleep apnea	3	0
Urological diseases	0	2
Other	1	5
Any background illness n (%)	14 (74%)	14 (70%)
**Dopaminergic antiparkinsonism agents:**	n (%)	n (%)
Levodopa	0	15 (75 %)
Carbidopa	0	11 (55 %)
Ropinirole	0	7 (35 %)
Pramipexole	0	6 (30 %)
Benserazide	0	4 (20 %)
Entacapone	0	4 (20 %)
Selegiline	0	2 (10 %)
Rasagiline	0	2 (10 %)
Rotigotine	0	1 (5 %)
Any antiparkinsonian medication n (%)	0 (0%)	20 (100%)

Clinical features of healthy controls and patients with Parkinson's disease (PD) at the time of sample collection. In patients with PD, any background illness describes subjects with illnesses other than PD.

Table S2. Mean relative abundance at phyla level. Related to Figure 4.

**Phylum (faeces)**	**Control faeces (%)**	**SD**	**PD faeces (%)**	**SD**	**Phylum (gut microbiota-derived EVs)**	**Control EVs (%)**	**SD**	**PD EVs (%)**	**SD**
Bacteroidota	49.5	15.6	54.0	17.3	Firmicutes	43.8	16.2	49.1	21.6
Firmicutes	43.2	18.3	40.8	15.8	Bacteroidota	40.4	20.7	43.5	21.4
Proteobacteria	5.1	7.8	2.0	2.3	Actinobacteriota	8.4	7.1	5.3	6.3
Actinobacteriota	2.1	3.9	3.0	5.8	Proteobacteria	6.7	4.7	1.6	2.3
Verrucomicrobiota	0.1	0.2	0.1	0.3	Fusobacteriota	0.2	0.3	0.1	0.3
Fusobacteriota	0.0	0.1	0.1	0.3	Acidobacteriota	0.1	0.3	0.0	0.1
Synergistota	0.0	0.0	0.1	0.3	Verrucomicrobiota	0.1	0.2	0.1	0.1
					Spirochaetota	0.1	0.2	0.0	0.0

Gut microbiota composition and taxonomic composition of extracellular vesicle (EV) origin in faeces of healthy controls and patients with Parkinson's disease (PD). SD: standard deviation.

Table S3. Mean relative abundance at genera or otherwise lowest taxonomical level. Related to Figure 4.

Genus (Faeces)	Control faeces (%)	SD	PD faeces (%)	SD	Genus (Gut microbiota-derived EVs)	Control EVs (%)	SD	PD EVs (%)	SD
Bacteroides	29.6	17.8	27.4	17.0	*Bacteroides*	25.5	14.8	20.1	12.8
Alistipes	6.3	4.9	9.7	7.4	*Alistipes*	6.0	6.0	14.6	15.0
Faecalibacterium	6.8	5.4	2.7	2.4	*(o) Izemoplasmatales*	3.1	12.5	16.7	17.8
Christensenellaceae R-7 group	2.3	2.1	4.2	5.1	*Prevotella_9*	4.7	16.0	3.9	10.5
(c) Bacilli RF39	3.1	7.8	2.6	4.5	*Staphylococcus*	4.4	6.6	2.3	2.9
Prevotella 9	1.7	3.8	3.8	8.9	*Corynebacterium*	3.9	3.3	2.4	2.9
Parabacteroides	2.2	1.6	3.2	2.0	*Streptococcus*	3.8	3.4	2.4	2.9
Sutterella	3.8	7.8	1.3	2.0	*Cutibacterium*	3.5	4.0	2.5	2.9
(f) Rikenellaceae RC9 gut group	0.4	1.0	4.4	12.2	*(f) Ruminococcaceae*	0.7	0.7	4.7	12.7
(o) Izemoplasmatales	0.6	0.9	3.4	4.4	*Christensenellaceae R-7 group*	1.7	1.7	3.5	6.0
Subdoligranulum	2.4	2.8	1.5	1.6	*Faecalibacterium*	2.9	2.6	2.2	3.0
Streptococcus	1.2	2.4	2.5	4.4	*Lactobacillus*	2.7	4.4	1.8	5.4
(o) Clostridia UCG-014	1.1	1.7	2.4	4.0	*(o) Clostridia vadinBB60 group*	0.9	3.0	2.5	4.4
Oscillospiraceae UCG-002	1.5	1.4	1.8	2.3	*Parabacteroides*	1.2	0.9	1.5	1.4
Barnesiella	2.1	2.7	1.1	1.7	*(f) Oscillospiraceae (g) UCG-005*	1.1	0.9	1.4	2.2
(o) Clostridia vadinBB60 group	0.5	1.2	2.5	4.5	*Erysipelotrichaceae_UCG-003*	1.8	5.7	0.7	0.7
Erysipelotrichaceae UCG-003	2.3	3.3	0.7	1.2	*Odoribacter*	1.0	0.8	1.3	1.9
(f) Ruminococcaceae	1.0	1.0	1.9	2.3	*Subdoligranulum*	1.6	1.3	0.8	1.5
Odoribacter	1.8	1.7	1.0	0.7	*Sutterella*	1.3	1.3	0.7	1.4
Corynebacterium	1.0	2.3	1.7	3.5	*Lachnospiraceae NK4A136 group*	1.8	1.7	0.3	0.4
Lachnospiraceae NK4A136 group	2.1	1.7	0.5	0.7	*Anaerococcus*	1.6	1.7	0.4	0.5
Blautia	1.8	1.9	0.5	0.5	*(o) Rhodospirillales*	1.8	2.2	0.2	0.5
(f) Muribaculaceae	1.0	2.5	1.2	4.6	*Blautia*	1.3	1.8	0.6	0.9
Cutibacterium	1.0	1.7	1.1	2.8	*Roseburia*	1.7	2.1	0.2	0.3
Prevotellaceae NK3B31 group	2.1	5.3	0.1	0.2	*[Eubacterium]_siraeum_group*	1.4	4.6	0.1	0.2
(f) [Eubacterium] coprostanoligenes group	0.6	0.7	1.4	2.2	*Agathobacter*	0.8	1.3	0.6	1.5
Roseburia	1.4	1.9	0.3	0.4	*(o) Oscillospirales (f)UCG-010*	0.3	2.4	0.9	2.4
Ruminococcus	1.4	1.7	0.3	0.5	*Ruminococcus*	0.9	1.3	0.3	0.7
Oscillospiraceae UCG-005	0.9	0.8	0.6	0.8	*Bradyrhizobium*	1.2	1.8	0.0	0.1
Staphylococcus	0.5	1.0	1.0	2.2					
(o) Oscillospirales (f) UCG-010	0.4	0.8	0.8	1.4					
Paraprevotella	1.1	1.9	0.2	0.3					

Gut microbiota composition and taxonomic composition of extracellular vesicle (EV) origin in faeces of healthy controls and patients with Parkinson's disease (PD). Taxa with an abundance of 1% or higher present either in healthy controls or patients with PD are shown in the table. SD: standard deviation, c: class, o: order, f: family.

Table S4. Biological process gene ontology annotations of bacterial proteins. Related to figure 9.

Biological processes	Control EVs	PD EVs
aerobic respiration [GO:0009060]	21	7
amino acid metabolic process [GO:0006520]	426	172
bacterial-type flagellum-dependent swarming motility [GO:0071978]	8	16
biological process involved in symbiotic interaction [GO:0044403]	4	10
carbohydrate derivative transport [GO:1901264]	23	20
carbohydrate metabolic process [GO:0005975]	224	219
carbohydrate transport [GO:0008643]	83	55
carboxylic acid metabolic process[GO:0019752]	22	7
cellular component organisation [GO:0016043]	80	229
cellular process [GO:0009987]	58	133
cobalamin transport [GO:0015889]	207	350
electron transport chain [GO:0022900]	21	167
hormone catabolic process [GO:0042447]	9	27
lipid metabolic process [GO:0006629]	89	259
metabolic process [GO:0008152]	34	16
monoatomic ion transport [GO:0006811]	329	593
nucleic acid metabolic process [GO:0090304]	29	34
nucleotide catabolic process [GO:0009166]	115	138
polysaccharide catabolic process [GO:0000272]	57	38
primary metabolic process [GO:0044238]	26	2
protein folding [GO:0006457]	335	279
protein localisation [GO:0008104]	35	35
protein metabolic process [GO:0019538]	164	332
protein stabilisation [GO:0050821]	7	13
protein transport [GO:0015031]	328	316
regulation of cellular metabolic process [GO:0031323]	553	505
response to stimulus [GO:0050896]	193	314
small molecule metabolic process [GO:0044281]	5	26
transmembrane transport [GO:0055085]	2	120
bacterial-type flagellum-dependent cell motility [GO:0071973]	1	n/a
intracellular sequestering of iron ion [GO:0006880]	70	n/a
carbohydrate derivative metabolic process [GO:1901135]	n/a	17
sporulation resulting in formation of a cellular spore [GO:0030435]	n/a	2

Values represent the number of protein hits derived from gut microbiota-derived extracellular vesicles (EVs) in healthy controls and patients with Parkinson’s disease (PD). Coverage was 18% for healthy controls and 16% for patients with PD. Annotations were retrieved from Uniprot. n/a: not applicable.

Table S5. Molecular function gene ontology annotations of bacterial proteins. Related to Figure 9.

Molecular function	Control EVs	PD EVs
2 iron, 2 sulphur cluster binding [GO:0051537]	21	7
amino acid binding [GO:0016597]	n/a	2
antioxidant activity [GO:0016209]	n/a	4
aspartyl esterase activity [GO:0045330]	3	n/a
ATP-dependent activity [GO:0140657]	225	230
carbohydrate binding [GO:0030246]	163	109
carbohydrate derivative binding [GO:0097367]	98	219
carbohydrate transmembrane transporter activity [GO:0015144]	88	257
cation binding [GO:0043169]	2	n/a
electron transfer activity [GO:0009055]	21	47
heme binding [GO:0020037]	n/a	40
hydrolase activity [GO:0016787]	715	862
isomerase activity [GO:0016853]	255	170
ligase activity [GO:0016874]	10	13
lipid binding [GO:0008289]	87	236
lyase activity [GO:0016829]	37	44
metal ion binding [GO:0046872]	283	190
nucleic acid binding [GO:0003676]	22	19
nucleotide binding [GO:0000166]	688	595
oxidoreductase activity [GO:0016491]	454	372
polysaccharide binding [GO:0030247]	92	181
protein binding [GO:0005515]	356	312
protein folding chaperone [GO:0044183]	1	1
protein-macromolecule adaptor activity [GO:0030674]	1	n/a
serine-type endopeptidase inhibitor activity [GO:0004867]	10	10
structural molecule activity [GO:0005198]	482	331
transferase activity [GO:0016740]	81	77
transmembrane transporter activity [GO:0022857]	544	n/a
transporter activity [GO:0005215]	n/a	1178

Values represent the number of protein hits derived from gut microbiota-derived extracellular vesicles (EVs) in healthy controls and patients with Parkinson's disease (PD). Coverage was 24% for healthy controls and 21% for patients with PD. Annotations were retrieved from Uniprot. n/a: not applicable.
